# Advances in ICP‐MS‐Based Nanoparticle Characterization: Techniques and Challenges in Biological Sample Analysis

**DOI:** 10.1002/jssc.70259

**Published:** 2025-09-10

**Authors:** Filip Gregar, Daniel Baron, Tomáš Pluháček

**Affiliations:** ^1^ Department of Analytical Chemistry, Faculty of Science Palacký University Olomouc Olomouc Czech Republic

**Keywords:** biological samples | inductively coupled plasma mass spectrometry (ICP‐MS) | nanoparticles | separation | single particle

## Abstract

The increasing use of engineered nanoparticles (NPs) in consumer and biomedical products has raised concern over their potential accumulation, transformation, and toxicity in biological systems. Accurate analytical methods are essential to detect, characterize, and quantify NPs in complex biological matrices. Inductively coupled plasma mass spectrometry (ICP‐MS) has emerged as a leading technique due to its high sensitivity, elemental selectivity, and quantitative capabilities. This review critically evaluates recent advances (from January 2020 onward) in ICP‐MS‐based methods for analysis of NPs in biological samples. Two main strategies are discussed: single‐particle ICP‐MS (spICP‐MS) and hyphenated techniques coupled to ICP‐MS. spICP‐MS allows direct determination of particle size, concentration, and metal content at environmentally relevant levels. It is the most widely used approach and is therefore examined in greater detail, with attention to extraction procedures, particle types, sample matrices, and inherent limitations. Advances in laser ablation spICP‐MS for tissue imaging and spatially resolved NPs detection are also covered. Methods using hyphenated techniques, such as hydrodynamic chromatography, size‐exclusion chromatography, capillary electrophoresis, Taylor dispersion analysis, and field‐flow fractionation, are increasingly employed to address limitations spICP‐MS. These approaches can provide enhanced insight into particle size distributions, aggregation behavior, and interactions with complex sample matrices. This review offers a comparative evaluation of both single‐particle and hyphenated methods, discussing their respective advantages and limitations. Emphasis is placed on the complementarity of these techniques and how their combined use can offer a more complete understanding of NPs’ fate in biological systems.

## 1 Introduction

Over the past decades, the outstanding properties of nanoscale materials have attracted worldwide interest and created a hotspot for rapidly growing basic and applied research, including nanomedicine. The most studied objects are nanoparticles (NPs) and nanoclusters (NCs). NCs represent nanostructures composed of a few to tens of atoms with a dimension of 1–10 nm [1]. Nowadays, the pivotal applications of NPs and NCs range from potent antibacterial and antiviral agents, contrast imaging agents, and unique drug delivery carriers with enhanced therapeutic efficacy to photothermal anticancer therapies [2, 3]. On the other hand, the exposure of the human body toward either applied or self‐produced NPs (e.g., mechanical tribocorrosion of implants) has raised regulatory‐derived concerns about their chemical state, toxicity, bioaccumulation, and negative impact on living organisms from cell cultures to animals or humans. The comprehensive characterization of nanoscale material parameters such as chemical composition, size, size distribution, shape, NP concentration, agglomeration/aggregation status, formation of protein corona, and other optical, physical, and magnetic properties is not possible without the logical combination of several techniques, like scanning electron microscopy (SEM), transmission electron microscopy (TEM), dynamic light scattering (DLS), inductively coupled plasma mass spectrometry (ICP‐MS), single‐particle inductively coupled plasma mass spectrometry (spICP‐MS), hydrodynamic chromatography ICP‐MS (HDC‐ICP‐MS), field flow fractionation ICP‐MS (FFF‐ICP‐MS), and capillary electrophoresis ICP‐MS (CE‐ICP‐MS) [4, 5, 6]. In contrast to NP synthesis, nanomedicine and nanomedomics have so far mainly focused on tracking the fundamental properties of NPs in biological materials. Such tracking by well‐developed techniques is limited by the complexity (mixture of NPs and other colloids, substances) and corrosive properties of prepared liquid NP suspensions/extracts.

On the other hand, there is a never‐ending quest to develop superior analytical techniques that allow advanced in situ characterization of metallic and metal oxide NPs in different biological matrices without laborious sample preparation that alters the nature of the incorporated NPs. Element/isotope‐specific ICP‐MS‐based approaches, such as single‐particle methods or hyphenation with separation techniques (e.g., CE‐ICP‐MS, FFF‐ICP‐MS, size‐exclusion chromatography [SEC‐ICP‐MS], and HDC‐ICP‐MS), are among the most promising analytical tools for studying metal‐based nanomaterials. The primary assumption for the accurate spICP‐MS characterization is a sufficient sample dilution to ensure continuous introduction of individual NPs, which generate a well‐differentiated discrete pulse of ions for the desired *m/z* ratio, acquired with an integration time starting from µs. However, the Achilles’ heel of spICP‐MS is the coexistence of the ionic forms (possible artifact from the NP extraction) of elements in the extracts, making the detection of small NPs challenging. Therefore, the adoption of the separation step (HDC, high‐performance liquid chromatography [HPLC], CE, and FFF) has been proposed as essential for the reliable NP characterization in biological samples containing a mixture of free ions and NPs fractions. Moreover, the recent developments have led to the introduction of laser ablation spICP‐MS (spLA‐ICP‐MS) and Taylor dispersion analysis (TDA‐ICP‐MS), opening a new window for the in situ characterization of NPs in various biological samples without altering the nature of NPs.

**FIGURE 1:**
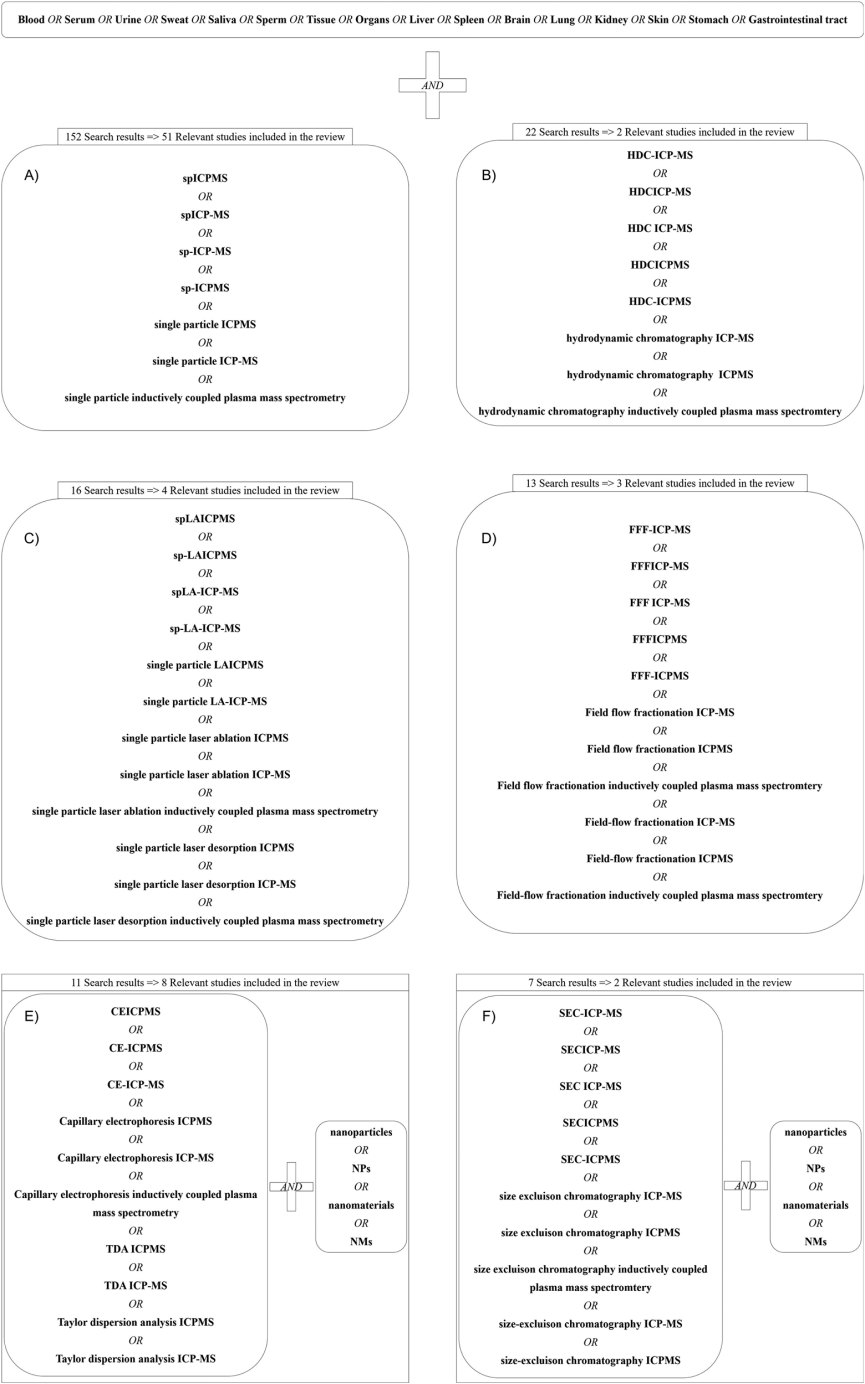
Overview of systematic literature searching 2020–2025 using Web of Science. The search was conducted in January 2025, with an additional search conducted in May 2025. Keywords related to specific analytical techniques were combined with specific biological matrices to identify relevant studies (the diagram shows the search being conducted in the opposite direction for visual clarity). The diagram is divided into six sections, each corresponding to a distinct technique: (A) spICP‐MS, (B) HDC‐ICP‐MS, (C) spLA‐ICP‐MS, (D) FFF‐ICP‐MS, (E) CE‐ICP‐MS or TDA‐ICP‐MS, and (F) SEC‐ICP‐MS. The last two were also combined with specific keywords to focus the search on nanoparticle detection. For each technique, the total number of articles retrieved from Web of Science is shown along with the number of studies ultimately included in the review.

The overall aim of the present review article is to systematically summarize the current state of the art of the ICP‐MS‐based NP analysis in biological matrices, focusing mainly on human and animal samples. The review process (Figure 1) was carried out to identify relevant studies available on the Web of Science (WOS) since January 2020 (WOS search was conducted in January 2025, with an additional search conducted in May 2025). The search was performed in several separate steps according to the technique used for NPs tracking: (1) studies targeting spICP‐MS; (2) studies targeting spLA‐ICP‐MS; (3) studies targeting CE‐ICP‐MS and TDA‐ICP‐MS; (4) studies targeting FFF‐ICP‐MS (although only asymmetric flow field‐flow fractionation (AF4) mode is used with ICP‐MS, the broader term FFF‐ICP‐MS was applied in the literature search as it increased the number of search results); and (5) studies targeting the hyphenation of liquid chromatography with ICP‐MS, from which the only relevant modes found were HDC and SEC. This resulted in a total of 221 articles dealing with the detection and characterization of NPs in biological samples by ICP‐MS‐derived techniques, of which only 70 were identified as relevant to the current review focused on the biological sample processing, respectively. These studies are critically evaluated, and the prospects of ICP‐MS‐based armories are discussed in the never‐ending hunt for the detection of the smallest NPs under the native conditions.

## 2 Single‐Particle Inductively Coupled Plasma Mass Spectrometry

**FIGURE 2:**
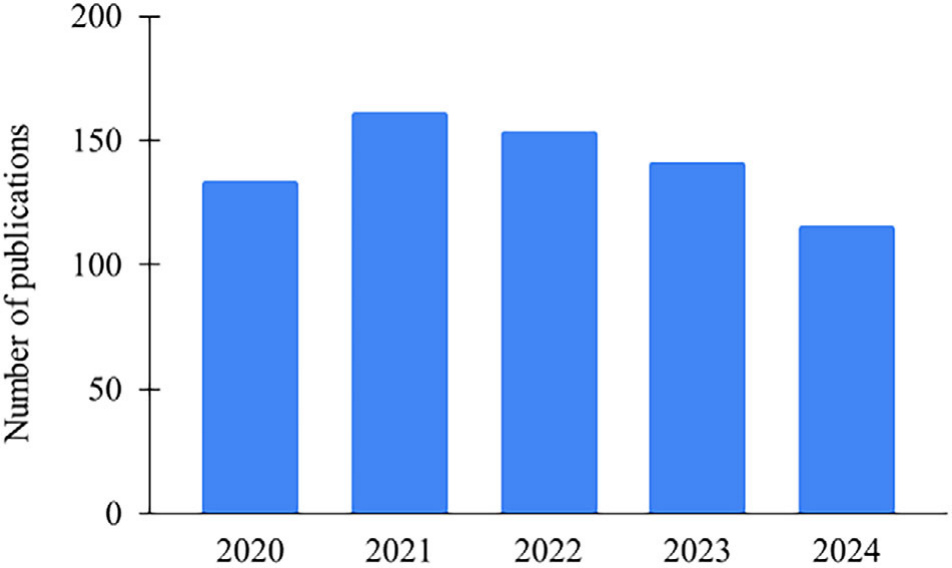
Number of publications found on the Web of Science in years 2020–2024. (Search: spICPMS OR spICP‐MS OR sp‐ICP‐MS OR sp‐ICPMS OR single‐particle ICPMS OR single‐particle ICP‐MS OR single particle inductively coupled plasma mass spectrometry.)

In recent years, spICP‐MS has shown great potential to become a strong tool in laboratories dealing with the analysis and characterization of nanomaterials, particularly metallic and metal oxide NPs. A potential that is nevertheless slowed down by several issues that need to be resolved before spICP‐MS could be considered a standalone technique for particle core size (=physical diameter, excluding any surface coatings, functional groups, or hydration layers), size distribution, and particle number concentration determination. However, the advantage of straightforward single NP detection and characterization in various kinds of sample types resulted in a published work approximately every three days in the last 5 years (Figure 2). The technique works by introducing a highly diluted NP suspension into the plasma discharge, where each particle is atomized and ionized. The resulting transient ion signals, or “pulses,” correspond to individual particles, with the intensity of each pulse being proportional to the mass of the NP, and the frequency of these pulses is directly related to the particle concentration. By calibrating with defined NP standards (mostly gold NP suspension) and optimizing parameters like sample uptake, transport efficiency, and time of analysis, sp‐ICP‐MS allows for the determination of NP core size (assuming particle composition, density, and shape—mostly spherical), size distribution, and particle number concentration [7]. The recent progress in the spICP‐MS technique and instrumentation, including basic principles of spICP‐MS operation, behavior of NPs in plasma discharge, and instrument design (ion optics, analyzers, detectors, etc.), together with a comparison of key features of different mass analyzers, has been reviewed in 2021 by Bolea et al. [8] and 2022 by Laycock et al. [9]. This review builds upon these reviews in a far simpler way. However, it broadens its scope outside single‐particle method, offering alternatives for ICP‐MS‐based NP analysis of biological samples.

The frequent use of spICP‐MS technique has increased the demand for sensitive, accurate, interference‐free, and rapid characterization of NPs and has prompted manufacturers to introduce a variety of ICP‐MS systems capable of single‐particle analysis. From all of the reviewed studies, 19 studies used instruments from PerkinElmer, 17 from Agilent Technologies, and 17 from Thermo Scientific, showing an even divide in instrument manufacturers across research laboratories. Considering the mass analyzers: 34 single‐quadrupole (SQ) from Agilent Technologies, PerkinElmer, and Thermo Fisher; 13 triple quadrupoles (TQ) from Agilent Technologies and Thermo Fisher; 3 time‐of‐flight analyzers (TOF) from TOFWERK and NU Instruments; and 1 sector field (SF) analyzer from Thermo Finnigan were used throughout the reviewed studies (Table 1). Despite the availability and accessibility of SQ ICP‐MS, the use of TQ systems is self‐explanatory in this regard, as it provides the advantage of stronger suppression of polyatomic interferences. TOF instruments seem to be the most auspicious with their multielement detection capability, which promises almost simultaneous detection of multiple particle species within a single analysis [10]. However, as seen from the literature search, their usage is yet not widespread. This is a result of the price, instrument availability, harder instrument operation, and harder data processing.

**TABLE 1:** Results of the literature search for studies where single‐particle inductively coupled plasma mass spectrometry (spICP‐MS) was utilized in the analysis of biological matrices (mainly human and animal).

Analyzed particles (size)	Sample matrix	Extraction methodology	MS instrument manufacturer	Mass analyzer	Smallest detectable particle	Ref.
**Enzymatic extraction**						
Ag (15–18.5 nm)	Freshwater amphipod	Dried animals were gently pressed and mixed with 10 mL of the digestion solution (45 mg/L proteinase K in buffer solution + 0.5% SDS + 50 mM NH_4_HCO_3_, pH adjusted to 8.0–8.2). Incubation for 3 h at 50°C and 100 RPM. The incubated digestion solution was filtered	Agilent Technologies	QQQ	10 nm	[28]
Ag (20 and 40 nm)	Ground beef	0.01–0.5 g of sample mixed with 5–8 mL extraction solution (1.5 mg/mL protease and Lipase in 5 mM HEPES buffer, pH 7.5). Ultrasonication for 15 min in an ice bath	Perkin Elmer	QQQ	10–12 nm	[11]
Ag (40 nm) Au (3, 60, and 100 nm) TiO_2_ (80, 100, and 150 nm)	Mussels	5 Enzymatic extractions compared. The best results were with a mixture of porcine pancreatin and lipase and centrifugation at 35°C for 12 h	Perkin Elmer	Q	NA	[29]
Ag TiO_2_	Mollusc	TiO_2_: 1 g of sample with 7.5 mL enzymatic solution (pancreatin and lipase 3 g/L), ultrasonication 10 min Ag: 1 g of sample with 10 mL enzymatic solution (pancreatin and lipase 2 g/L), ultrasonication 10 min	Perkin Elmer	Q	NA	[33]
Au (30 and 60 nm)	Mussels	1 g of homogenized mussel sample, 3 mL of protease enzyme solution was added prior to incubation with heat‐shaker at 300 RPM and 50°C for 1 h	Agilent Technologies	Q	18 nm	[30]
CeO_2_ (30–50 nm)	Rat liver	4 mg lyophilized and grounded sample mixed with 1 950 µL enzyme solution (0.02% proteinase K, 0.5% SDS, and 0.08% EDTA in 10 mM Tris–HCl buffer, pH 7.4). Incubation at 37°C overnight shaking at 100 RPM	Agilent Technologies	Q	NA	[12]
CeO_2_ (30–50 nm)	Mouser liver, spleen, thymus and kidney	Same methodology as [12]	Agilent Technologies	Q	NA	[13]
HgSe	Raptor liver	20 mg of sample was defatted. Extraction by solution (1 mg/mL protease and 5 mg/mL SDS in 50 mM ammonium carbonate buffer, pH 7.4) overnight at 37°C	Agilent Technologies	Q	17.6 nm	[14]
HgSe (200–770 nm)	Sperm whale liver	20 mg of dry liver was incubated overnight at 37°C with extraction solution (1 mg/mL proteinase K and 5 mg/mL SDS, the solution was buffered to pH 7.4 with 50 mM ammonium bicarbonate) and finally diluted in Milli‐Q water	Nu Instruments	TOF	NA	[15]
HgSe	Petrel seabird liver, kidney, muscle, brain, feathers, and blood	25 mg freeze dried sample was defatted, mixed with 3 mL of solution (2 mg/mL proteinase K in 50 mM ammonium bicarbonate buffer, pH 7.4) and ultrasonicated for 2 min. Then 2 mL of 4% SDS was added before 1 h of ultrasonication	Agilent Technologies	Q	30 nm (Se detection) 46 nm (Hg detection)	[16]
SiO_2_ (14–23 nm and 13–45 nm)	Rat liver	Liver homogenates were sonicated for 5 min in 50 mM Tris–HCl buffer (pH 8) with 10% SDS. Samples were incubated with 2 mg/mL proteinase K (45°C, 1 h, agitation), sonicated again (5 min)	Perkin Elmer	Q	350 nm	[18]
SiO_2_ (50–500 nm) TiO_2_ (50–500 nm)	Human liver, spleen, kidney and intestine tissue	Proteinase K and digestion buffer were added to the sample, and the tube was incubated for 16 h at 37°C	Thermo Finnigan Agilent Technologies	SF QQQ	NA	[58]
Ti particles induced from Ti‐6Al‐4V dental implants	Mouse liver, spleen, brain, lungs, and blood	25 mg dried and milled tissue sample mixed with 1.5 mL enzymatic solution (3 mg/mL of proteinase K, 0.5% SDS, 10 mmol/L TRIS buffer, pH 7.4–8.2), 1 h sonication, overnight shaking at 37°C	Thermo Fisher	QQQ	NA	[17]
TiO_2_ (21 nm)	Zebrafish	10 mg of tissue mixed with 0.2 mL of water, then sonicated for 10 min in an ice water bath. 1 mL of enzyme solution (0.05% w/v proteinase K, 50 mM ammonium bicarbonate, and 0.05% w/v SDS) was added and incubated at 50°C for 3 h in a water bath	Perkin Elmer	Q	48.6 nm	[31]
TiO_2_ (130 nm)	Mussels	200 mg of sample mixed with 3.09 mL digestion buffer (600 mg of Tris buffer in 200 mL of Milli‐Q water) and 910 µL of enzyme solution (37.5 mg of proteinase K was dissolved in 50 mL of Milli‐Q water) and incubated/shaken for 3 h at 37°C in a water bath	Thermo Fisher	Q	NA	[32]
**Alkaline extraction**						
Ag (35.7–55.5 nm)	Mouse brain and liver	Tissue samples were mixed with 20% TMAH at a ratio of 20:1 (mL/g) for 24 h at room temperature. The digested solutions were diluted 100‐fold in 0.1% Triton X‐100 solution (prepared in Milli‐Q water) before measurement	Agilent Technologies	QQQ	22.3 nm	[19]
Ag (100 nm)	Mouse blood and skin	Homogenized samples were mixed with 0.1 mol/L NaOH at v/v ration 1:1 and incubated at 37°C for 3 h	Agilent Technologies	Q	NA	[20]
Ag (20 and 40 nm)	Ground beef	5 mL of TMAH solution (10% or 2.5%) to 0.02 g of lyophilized sample, followed by 15 min of ultrasonication	Perkin Elmer	QQQ	10–12 nm	[11]
Ag (10, 20, 40, and 60 nm)	Pig and chicken feces	100 mg feces were mixed with 2 mL TMAH (25%) and 0.4 mL cysteine (0.5%), and tumbled for 24 h at 28 RPM in darkness	Perkin Elmer	Q	NA	[56]
Ag (10, 20, 40, and 60 nm)	Pig and chicken feces	500 mg feces were treated with 10 mM TSPP, pH adjusted with 2 mM NaOH. Samples were shaken (200 RPM), ultrasonicated (30 min), and centrifuged (10 min at 157 g)	Perkin Elmer	Q	NA	[56]
Ag	Snails	Snail soft tissues (0.02 g) were digested with 20% TMAH. After samples were shaken at 400 RPM and 70°C for 24 h, accompanied by 30 min of sonication every 12 h	Perkin Elmer	Q	11 nm	[27]
Ag (75 nm) Ag‐PEG coated (41 nm)	Seronorm blood	0.6 mL of 10%–25% TMAH was added per 0.1 g of blood, 5 min sonication, and 24 h incubation at room temperature in the dark. Final dilution to 6 g with 0.1% Triton X‐100	Agilent Technologies	QQQ	11 nm	[47]
Ag‐derived particles (from 30 to 60 nm)	Rainbow trout hind intestine, liver and kidney, and whole carcass	20 mg of tissue mixed with extraction solution of 20% TMAH containing 5 mmol/L CaCl_2_. The samples were left overnight at room temperature in a dark, dry storage cupboard	Thermo Fisher	Q	14 nm	[34]
Ag (100 nm) Au (60 nm)	Ground beef	Multiple Alkaline extractions were tested. The optimal methodology was chosen as 1.75 g of sample mixed with 35 mL of 20% TMAH	Perkin Elmer	Q	NA	[24]
Ag (20, 60, and 100 nm) Au (5, 20, 40, and 60 nm)	Human blood	Blood was extracted using a 1:5 ratio of TMAH, 25% to blood, sonicated for 1 h in an ice‐cold water bath, left at room temperature for 24 h	Thermo Fisher	Q	NA	[42]
Ag (29 nm) Au (15 nm) In_2_O_3_ (38 nm) TiO_2_ (84 nm) Ir, Pd, Pt	Human blood	Blood was extracted using a 1:5 ratio of TMAH, 25% to blood, sonicated for 1 h in an ice‐cold water bath, left at room temperature for 24 h	Thermo Fisher	Q	NA	[40]
Ag (40 nm) Au (3, 60, and 100 nm) TiO_2_ (80, 100, and 150 nm)	Mussels	2 alkaline extractions compared. The best results were achieved with: dry sample, 5% TMAH at 25°C for 12 h	Perkin Elmer	Q	NA	[29]
Au (30 and 60 nm)	Chicken tissue Mouse heart, liver, spleen, lungs, and kidney	0.2 g tissue, 500 µL of digestion buffer and pure water were added into the tube. After tissue grinding, the tissues were digested with 2% TMAH for 10 min	Perkin Elmer	Q	NA	[21]
Au (48 and 88 nm)	Mouse heart, liver, spleen, lungs, kidneys, intestines, brain, and blood	Tissue samples were ground with Triton X‐100 digestion buffer, and a homogenous sample was repeatedly washed with 2% TMAH (25:1 solvent to sample) Blood samples were directly mixed with 2% TMAH	NA	NA	NA	[22]
Au (47 and 87 nm)	Mouse heart, liver, spleen, lungs, kidneys, intestines, aorta, and thymus	2–4 mL buffer was added to the chopped tissue before grinding. After 10 min of grinding, TMAH at 2% concentration was added	Perkin Elmer	Q	NA	[23]
Au (80, 100, and 150 nm)	Nematodes	0.50–1 mg samples of lyophilized nematodes were treated with 1 mL of 7% (volume fraction) TMAH. Samples were vortexed for 30 s at room temperature for 2 h	Thermo Fisher	Q	NA	[35]
Au (35–55 and 30–65 nm) Another 20 metals	Oyster and clams	0.1 g of wet seafood tissue was mixed with 2 mL of 20% TMAH for alkaline digestion. The mixture was vortexed, sonicated for 60 min at 37°C, and further shaken at 80 RPM for 24 h at room temperature	Agilent Technologies	QQQ	NA	[36]
CeO_2_ (30–50 nm)	Mouse liver, spleen, thymus, and kidney	4 mg of lyophilized tissue was digested with 2 mL of 20% TMAH solution. The samples were vortexed to mix thoroughly and left at room temperature overnight	Agilent Technologies	Q	NA	[13]
CuO (25 nm)	Planktonic crustacean Mysid shrimp	Samples mixed with 1 mL 20% TMAH, water bath sonication 30 min, incubation 24 h at 70°C at shaking speed 800 RPM, water bath sonication 30 min	Perkin Elmer	Q	NA	[37]
Fe‐derived ultrafine particles	Mouse blood, heart, liver, spleen, lung, kidney, and brain	20% TMAH mixed with tissue in a 1:20 ratio for 24 h at room temperature, digest treated by cyclic magnetic extraction	NA	NA	NA	[25]
Hg‐derived particles	Cetaceans liver and muscle	20% TMAH 20:1 solvent to sample ratio, 12 h at room temperature in dark, 1 h sonication. pH neutralization with HN0_3_, diluted with 2% FL‐70 before analysis	Agilent Technologies	QQQ	NA	[71]
Pt (50 and 70 nm)	Human urine and blood serum	TMAH 1% mixed with samples was ultrasonicated for 1 min to disperse PtNPs. Samples were left for 24 h at 4°C	Thermo Fisher	Q	21.63 nm	[41]
ZnO (61.3–78.6 nm)	Canned seafood (tuna, mackerel, anchovy and clam)	0.25 g, wet weight sample in an ultrasonic bath with 5 mL of TMAH (20% v/v) for 30 min at 37°C, and another 24 h at room temperature	Perkin Elmer	Q	27 nm	[38]
**Acid extraction**						
CuO spherical (<50 nm) CuO rod shaped (10–12 nm × 17–100 nm)	Rainbow trout liver, intestine, stomach, gill, muscle, and brain	The tissue samples were digested using 30% H_2_O_2_ for 1 h at 80°C in a water bath. The dispersions were immediately diluted with Milli‐Q water and sonicated for 3–5 min using ultrasonic bath	Perkin Elmer	Q	NA	[39]
HgSe	Petrel seabird liver, kidney, muscle, brain, feathers, and blood	25 mg homogenized freeze‐dried sample defatted was mixed with 5 mL of 50% formic acid kept at 85°C for 2 h in a hot block	Agilent Technologies	Q	30 nm (Se detection) 46 nm (Hg detection)	[16]
TiO_2_ (39–187 nm)	Human periprosthetic tissue	MW assisted digestion of tissue: 50 mg of lyophilized tissue, 4 mL ultrapure water, 1.5 mL HNO_3_, 0.5 mL H_2_O_2_	Agilent Technologies	QQQ	NA	[59]
TiO_2_	Snails	Snail soft tissues (0.02 g) were digested with 35% H_2_O_2_. After samples were shaken at 400 RPM and 70°C for 24 h, accompanied by 30 min of sonication every 12 h	Perkin Elmer	Q	44 nm	[27]
**Dilution**						
Ag PVP coated (20, 50, and 100 nm)	Artificial sweat (multiple guidelines used for preparation)	Each 1:10 dilution of the mixtures was diluted further 1:10 000 with ultrapure water to a minimum of 5 mL for spICP‐MS measurement	Agilent Technologies	Q	NA	[50]
Ag (20, 60, and 100 nm) Au (5, 20, 40, and 60 nm)	Human urine and plasma	Urine and serum samples were diluted 1:10 with ultrapure deionized water	Thermo Fisher	Q	NA	[42]
Ag (29 nm) Au (15 nm) In_2_O_3_ (38 nm) TiO_2_ (84 nm) Ir, Pd, Pt	Human urine	Urine samples were diluted 1:10 with ultrapure deionized water	Thermo Fisher	Q	NA	[40]
Ag (40 nm) TiO_2_ (60 nm) ZnO (30 nm)	In vitro digestion fluids used to digest canned seafood (tuna)	Samples were digested sequentially with α‐amylase (37°C, 2 min, pH 7), pepsin (120 min, pH 3), and pancreatin with bile salts (120 min, pH 7) at 37°C. pH was adjusted with 1 M HCl and NaOH. Diluted samples (1:10) were sonicated for 20 min	Perkin Elmer	Q	37 nm TiO_2_ 19 nm Ag 29 nm for ZnO	[48]
Au (50 nm)	Mouse blood	Blood samples were diluted to appropriate concentrations using ultrapure water	Agilent Technologies	QQQ	19 nm	[57]
Au (50 nm)	Human blood and urine	Dilution of samples directly by clinical diluent (0.5 g of EDTA, 10 mL isopropyl alcohol, 10 mL ammonia solution, 0.5 g Triton X‐100, and filled up to 1 L Milli‐Q water)	Perkin Elmer	QQQ	NA	[43]
Carbon coated FeC_3_	Bovine whole blood, heparin anticoagulated	Dilution by ultrapure water	TOFWERK	TOF	NA	[49]
Cr_2_O_3_ (60 nm) Mn_3_O_4_ (30 nm) NiO (18 nm)	Human exhaled breath condensate, urine, plasma	Dilution steps with ultrapure deionized water. After each dilution step, samples and standards were vortexed for 1 min and sonicated for 10 min using an ultrasonic ice‐cooled water bath	Thermo Fisher	Q	8.2–25.1 nm Cr_2_O_3_ 15.6–29.9 nm Mn_3_O_4_ 17.6–25.1 nm NiO	[44]
Pt (30 and 50 nm)	Human urine	The samples were prepared freshly before the analysis and by dilution in UPW 1:10	Thermo Fisher	QQQ	22.7 nm	[45]
TiO_2_ (71–112 nm)	Human urine	Urine samples were sonicated in an ultrasound bath for 5 min; then, 125 µL was diluted in 10 mL of 0.1% HNO_3_ to achieve a dilution factor of 1:80 (urine:diluent)	Perkin Elmer	Q	44 nm	[67]
TiO_2_ (25 nm)	Artificial saliva	Dilution water (at least 1000 times)	Thermo Fisher	QQQ	NA	[51]
TiO_2_ (70 nm)	Human blood and urine	Aliquots of 300 µL were diluted to a final volume of 10 mL with water	Perkin Elmer	Q	42 nm	[46]
**Other extraction methods**						
CuO	Artificial lung fluid (gambles solution)	Direct measurement in matrix media	Thermo Fisher	Q	NA	[52]
Fe‐ and Ti‐containing NPs from coal flying ash	Artificial lung fluid (gambles solution)	Direct measurement in matrix media	Perkin Elmer	Q	NA	[53]
Ag (10, 20, 40, and 60 nm)	Pig and chicken feces	Water extraction: 100 mg of ground feces was mixed with 1 mL ultrapure water (1:10 solid/reagent ratio). The suspensions were shaken at 28 RPM for 4 h at room temperature in darkness, sonicated for 5 min, rested for 20 min, and centrifuged at 7000 *g* for 5 min at 21°C	Perkin Elmer	Q	NA	[56]
HgSe (200–770 nm)	Sperm whale liver	Water extraction: 20 mg of whale liver tissue was incubated overnight at 15°C in a solution of 5 mg/mL SDS prepared in Milli‐Q water. The samples were vortexed for 15 s before incubation	Nu Instruments	TOF	37 nm TiO_2_ 19 nm Ag 29 nm for ZnO	[15]
Ag (40 and 70 nm)	Artificial sweat leached in commercial clothing	The artificial sweat solution was prepared according to ISO 105‐E04. For leaching experiments, the textile‐to‐solution ratio was 1:50 (1 g textile to 50 mL solution). Extraction was carried out at 37°C in a shaking water bath for 30 min	Agilent Technologies	Q	17 nm	[54]
TiO_2_ (60–260 nm)	Artificial sweat	The artificial sweat solution was prepared according to ISO 105‐E04. Yarn segments (0.5 g, 40 mm) were wetted with 50 mL of sweat, and solutions were collected at various time points	Perkin Elmer	Q	NA	[55]
Gd‐derived particles	Rat brain (deep cerebellar nuclei)	Sample (∼50 mg) was homogenized in ammonium acetate (pH 7.4), centrifuged at 20 800 *g* for 30 min at 4°C, and the supernatant separated. The pellet was washed, centrifuged again, solubilized in urea, and the urea‐soluble fraction was stored. The urea‐insoluble fraction was washed, stored at −20°C, resuspended in water, diluted, and analyzed	Agilent Technologies	Q	NA	[107]

*Note*: The table is divided into sections according to the type of extraction used. It contains information on the type and size of analyzed particles, sample matrix, brief description of extraction methodology, MS instrument manufacturer, type of mass analyzer used and size the smallest detectable particle (where available).

Abbreviations: NA = not available; Q = quadrupole; QQQ = triple quadrupole; SF = sector‐field; TOF = time‐of‐flight.

This section offers an overview of 51 research studies where spICP‐MS was utilized for the analysis of NPs in biological samples in the last 5 years. The literature research aimed to establish the state of the art in terms of analyzed particles, sample matrix, and extraction procedure, while simultaneously dealing with the limitations of the technique [9].

### 2.1 Sample Preparation: Analyzed Matrices and Extraction Methods

**FIGURE 3:**
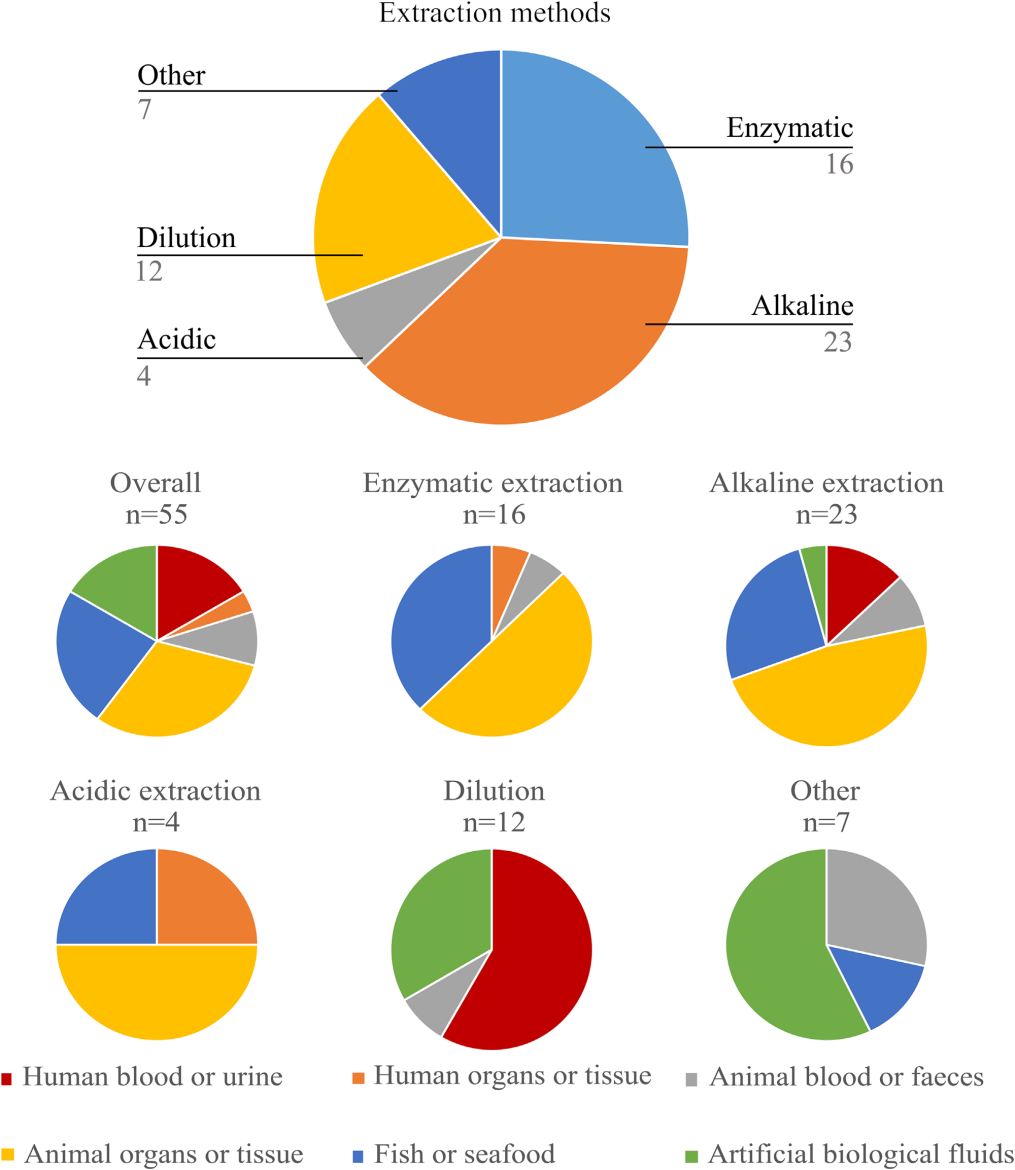
Summary of the extraction methods used for NPs release from biological samples. The chart at the top shows the distribution of extraction methods used in the reviewed literature. Charts at the bottom show the application of each digestion method across different sample matrices. Biological specimens have been divided into six categories: (1) human blood or urine; (2) human organs or tissue; (3) animal blood or feces (no animal urine found in the reviewed studies); (4) animal organs or tissue (mostly rat and mouse); (5) fish or seafood; and (6) artificial biological fluids (sweat, saliva, gastro fluids, Seronorm blood). The total number of studies does not equal the sum of individual extraction methods, as some studies employed multiple extraction techniques on the same sample, resulting in a slightly higher cumulative count for individual methods than the overall number of unique studies.

The preparation of well‐stabilized suspensions of NPs without significantly changing their character or even their dispersion is an essential step for the spICP‐MS analysis. It is worth mentioning that the biological specimens’ collection and storage could strongly influence the detection power and reliability of the obtained NPs’ characteristics [9]. Thus, the selection of proper extraction methodology significantly influences the efficiency of NP release, dissolution, agglomeration, sample stability, and overall analytical performance. Different extraction techniques vary in their applicability depending on the nature of the biological matrix and the stability of the NPs, with some methods being preferred for specific sample types. The summary of different extraction methods used with different sample matrices is in Figure 3. There has been sustained interest in identifying a versatile extraction methodology; however, no such methodology has been discovered yet (Figure 3). For the analysis of solid samples, there is almost an even divide between enzymatic and alkaline extractions, whereas for fluids, dilution is the most opted‐for option.

Up to date, animal organs or tissues were the most commonly analyzed matrices [11, 12, 13, 14, 15, 16, 17, 18, 19, 20, 21, 22, 23, 24, 25, 26, 27], showing the continued interest in understanding NP bioaccumulation, biodistribution, and potential toxicological effects in animal models. In 14 instances, fish or seafood was analyzed, reflecting the concern of contamination of the aquatic environment by the nanomaterial industry [15, 28, 29, 30, 31, 32, 33, 34, 35, 36, 37, 38, 39]. Human urine or blood was used in 12 studies, as it presents the perfect and less invasive way to assess the NPs exposure, their effects on metabolism, and toxicological influences on the human population [12, 40, 41, 42, 43, 44, 45, 46]. Artificial biological fluids (five instances) serve as control model systems for the simulation of physiological conditions in the human body [47, 48, 49, 50, 51, 52, 53, 54, 55]. As only five studies concerned the use of animal blood or feces, the focus seems to be rather on the whole‐body distribution of NPs [12, 16, 20, 56, 57]. Yet these samples are still relevant in establishing the NP uptake, circulation, and excretion in animal models. Human organs or tissues have been studied in only two cases [58, 59]. This limited research focus can be attributed to the ethical and practical challenges of obtaining human tissue samples. Unlike blood or urine, which can be collected noninvasively, organ and tissue sampling typically requires postmortem analysis or biopsy procedures, both subject to strict ethical regulations and availability constraints (Table 1).

Alkaline extraction was the most commonly used method, appearing in 22 studies with 23 different methodologies, and was applied to the widest variety of samples. Commonly, it was used with solid samples, appearing 17 times (11 times animal organs or tissue [11, 13, 19, 20, 21, 22, 23, 24, 25, 26, 27]; 6 times fish or seafood [29, 34, 35, 36, 37, 38]). However, it also found its uses in biological fluids as this approach was used three times with human blood or urine [40, 41, 42], two times with animal blood or feces [20, 56], and one time with artificial biological fluids [47]. All alkaline extractions were carried out primarily by tetramethylammonium hydroxide (TMAH), and rarely NaOH or tetrasodium pyrophosphate (TSPP) was used. The TMAH concentration ranges throughout studies from 1% to 25%; however, the most frequently used concentration was 20%. Generally, the samples are either vortexed, sonicated, or both before the incubation with the time of this homogenization ranging from 30 s to 1 h. The incubation itself was most commonly carried out over 24 h or overnight at room temperature. Nevertheless, short incubations taking 10 or 15 min [11, 21] and high temperatures up to 70°C [37] were also reported. Regularly, the extraction is ended with centrifugation for the removal of residual matrix and appropriate sample dilution prior to analysis. This widespread use of alkaline extraction suggests its relative simplicity and reliability in the digestion of biological matrices, while releasing almost intact particles, although there is still a potential risk of either particle dissolution or agglomeration in the alkaline particle suspension as the digestion mixture does not target specifically only the sample matrix.

Enzyme extraction was the second most utilized method being applied in 15 studies. It was predominantly used with solid samples with 8 times with animal organs or tissues [11, 12, 13, 14, 15, 16, 17, 18], 6 times with fish or seafood [28, 29, 30, 31, 32, 33], and 1 time each with human blood or urine [12], human organs or tissue [58], and animal blood or feces [17]. Proteinase K is unambiguously the most common enzyme being employed in 10 cases [12, 13, 15, 16, 17, 18, 28, 31, 32, 58]. The homogenization steps comprised either vortexing or sonication from 30 s to 1 h. The incubation was typically conducted overnight at 37°C. However, shorter incubation periods as brief as 15 min were reported [11] as well as elevated temperatures as high as 50°C [28, 30, 31]. The extraction generally ends with sample dilution before analysis, and sometimes, the sample is filtered as well. This approach is highly preferred because it targets specifically the organic matrix and usually leaves the particles intact. However, these extraction protocols are usually more complicated, requiring precise monitoring of temperature and pH. Moreover, enzymatic digestions tend to leave residual tissue after the incubation period, which brings its own drawbacks. Utilizing filtration or sedimentation techniques brings a potential for the removal of a chunk of analyzed particles or more contamination risks (Table 1).

Dilution was utilized in 12 studies. By its nature, it is only applicable to liquid samples, thus finding its uses primarily in the analysis of human blood or urine [40, 42–46] and artificial biological fluids [48–51], with only one case of analysis of animal blood or feces [57]. Usually, the NPs extraction is carried out using ultrapure water with a dilution ratio ranging from 1:10 to 1:100 000, depending on the sample matrix. The technique offers simple, straightforward sample preparation with minimal sample handling, thus making it suitable for liquid biological matrices in cases where no matrix effects appear (Table 1).

Acidic extraction is utilized rarely, appearing only four times in the review and only in connection with release from solid samples. The limited use highlights the danger of acidic dissolving and altering metallic particles. This disadvantage is often countered by using weaker acids (hydrogen peroxide [27, 39] or formic acid [16], with one study utilizing a diluted mixture of nitric acid [59]). The other time this extraction is usable is in the analysis of highly inert oxides, for example, TiO_2_, where one work utilizes a mixture of nitric acid and hydrogen peroxide to completely dissolve the organic matrix, leaving just the particles. In these specific cases, the potential for acidic extraction might be bigger than the number of studies using it might suggest. Proper method optimization to ensure satisfactory particle recovery with acids enables full utilization of the unrivaled advantage of microwave digestion, which significantly reduces extraction time compared to alkaline or enzymatic protocols that generally require overnight sample incubation. This could significantly increase the sample throughput for future routine analysis. However, for any other analytes, these extraction methods are unusable (Table 1).

The other category comprises extraction protocols that did not categorize into any of the abovementioned groups. In two cases, it was direct measurement in matrix media, where both were carried out in artificial lung fluid [52, 53]. Water dissolution/release was also used in another two cases. First, it was used to evaluate the potential release of particles from pig and chicken feces, so the best possible extraction was not the aim [56]. Second, it was compared with enzymatic extraction in the analysis of HgSe particles in whale liver and was deemed inferior [15]. Two more studies study the leaching of textiles in artificial sweat [54, 55]. Methods in this category are applicable only in specific situations, limiting their general use. It is worth mentioning that the development, optimization, and validation of the extraction protocol are often hampered by the lack of available certified reference materials with defined and similar chemical composition, size, and size distribution. Therefore, only a few studies aimed at the determination of the small particle solubilization [60, 61] or even the NPs size alteration.

Among the various extraction methods of NPs in biological matrices, alkaline and enzymatic extractions were the most widely employed (Figure 3). Alkaline extraction demonstrated versatility across a broad range of biological samples. Its popularity seems to be largely due to its simplicity, efficiency, and ability to digest complex biological matrices while preserving the integrity of NPs. TMAH was the dominant reagent, often used at 20% concentration, with overnight incubation at room temperature. However, despite its effectiveness, the risk of particle agglomeration in alkaline particle suspensions remains a potential drawback that must be considered in data interpretation. In contrast, enzymatic extraction, while also primarily applied to solid samples, offered a more targeted digestion process, with proteinase K being the enzyme of choice in most cases. The method's strength lies in its ability to selectively break down organic components while preserving NPs, making it particularly useful for delicate sample matrices, where minimizing structural alteration of the particles is critical. However, enzymatic extraction is more complex, requiring precise temperature and pH control, and has limited applicability on different matrices where an enzyme like lipase could be satisfactory for fatty matrices but can struggle digesting protein‐heavy matrices. Proteinase K is the other way around. These findings are in good agreement with the previously published review [9]. The slight difference is that it seems that the trend shifted more toward alkaline digestion. Some reasons for this were given in the paragraphs above. However, there is another aspect not previously discussed, and that is the economic aspect. At the time of writing this review, the price for 1 L of 25% TMAH is $208, whereas the price for 1 g of proteinase K is $1240 (prices pulled from https://www.sigmaaldrich.com/US/en). With the average TMAH consumption of 5 mL of 20% TMAH per sample, we arrive at roughly $0.8 per sample for the digestion agent. For proteinase K with the average enzyme concentration of 2 mg/mL and 2 mL of enzyme solution per sample, we can calculate that the digestion of one sample costs about $4.95 for just the enzyme. We are aware that this rough estimation omits multiple factors like different sample amounts and other costs like filters and stabilizer agents. However, it simply illustrates another potential reason for researchers to favor the alkaline extractions more. Moreover, when Sun et al. [29] compared these two approaches, they found that the alkaline extraction offered superior digestion recovery rates (the protocol with the highest yield for enzymatic extraction reached recoveries of 76% for Ag NPs and 74% for Au NPs, whereas the alkaline extraction protocol with the highest yield reached up to 82% for Ag NPs and 83% for Au NPs).

### 2.2 Analyzed Particles

**FIGURE 4:**
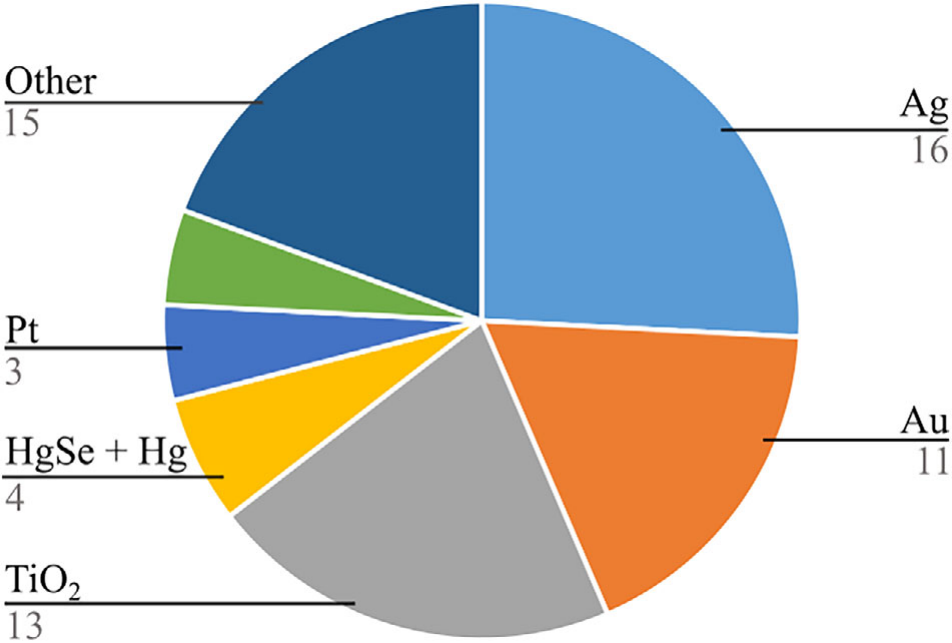
An overview of the nature of particles analyzed in the reviewed studies from Table 1.

Single‐particle ICP‐MS is versatile in its ability to analyze a wide range of metal‐containing particles. The direction of the field could be read from Figure 4, where almost 70% of the published works deal either with Ag, Au, or TiO_2_ NPs. This trend is the same one reported before [8, 9]. Silver and gold NPs have been extensively used due to their well‐defined properties and availability. A wide range of high‐quality options, including particle suspensions and powders with precisely controlled sizes and shapes, are commercially available, making them ideal for method development. Furthermore, the antibacterial effect of silver NPs earned them a varied spectrum of applications, namely, in the medical, pharmaceutical, cosmetic, and clothing industries [62]. Thanks to its inert and non‐toxic character, TiO_2_ nanopowder has been extensively used in the cosmetic, food, and medical industries. However, in recent years, multiple studies have pointed out potential health risks [63–66] connected with their use, to which the European Union reacted with a ban on E171 (titanium dioxide used in food processing) in January 2022. Thus, these particles have become the focus of intensive research. Moreover, their widespread use makes them readily accessible, making them well‐suited for validating the methodology. Only a few studies are focused on actual in situ studies examining these particles released into the environment due to anthropogenic activity. Most often, studies where Au, Ag, or TiO_2_ NPs were used dealt with two different types of experiments. First, the sample matrix (animal organs/tissue or human blood/urine) is mixed with NP standards ex vivo to validate or evaluate the efficiency of the extraction process (e.g., [11, 21, 29, 30, 36, 47, 67]). Second, animals are either fed, injected, or forced to breathe some form of NPs, and after animal euthanasia, the effects on the living organism are evaluated (e.g., [17, 20, 21, 27, 28, 31, 32, 34, 56]). The first approach is well‐suited for validating extraction methodologies, as it offers better control over particle size and experimental conditions; however, it does not truly reflect the methodology's ability to liberate particles from within biological tissue. In contrast, the second approach allows for the observation of the actual fate of NPs within a living organism, providing valuable insights, though the interpretation of the processes and transformations occurring in vivo remains challenging.

The next material, mercury, has been shown to accumulate in high concentrations in major organs of predatory animals and even to accumulate with age in whales [68, 69]. Ingested mercury has been shown to have the potential to form inert HgSe nanostructures. The effect on the organism is still debated. On one side, the formation of these nanostructures inhibits the toxicity of Hg in the system, whereas, on the other hand, the depletion of Se as an essential element may lead to indirect toxicity [70]. As the role and destiny of Hg in predators are still unclear, the analyses of Hg and HgSe‐derived particles are a point of interest for researchers trying to understand this phenomenon. Throughout the reviewed studies, HgSe particles were analyzed in birds [14, 16] and in cetaceans [15, 71].

Recently, Pt NPs have gathered attention in nanomaterial science, thanks to their unique structural and catalytic properties. Moreover, they have a great potential to become well‐sought‐after material in medical environments thanks to their ability to act as antibacterial, antifungal, anticancer, and so forth, agents. Nevertheless, their use is limited due to growing concerns about their potential health risks [72–74]. To properly assess their toxicity and health hazards, methods for analysis and characterization of Pt NPs are in demand. This review contains three studies dealing with Pt NPs. Two of these studies focus on the development of analytical methodology for Pt NPs detection in human blood or urine [41, 45]. The third study explored the exposure of nanomaterial workers to NPs [40].

Fifteen research works also contained other particles than those named above. CuO NPs have found their use in semiconductors, electronic chips, and so forth thanks to their superior thermophysical properties and relatively cheap manufacturing [75]. Of the three reviewed studies, two explore the bioaccumulation in aquatic organisms [37, 39]. One study compared CuO NPs detection in different matrix media with different extraction processes. CeO_2_ NPs are a component of multiple consumer products, namely, automobile catalyzers, ceramics, and gasoline. Its increasing application has led to gradual exposure to the environment, and questions regarding its health risks have risen. As such, detection tools have become necessary [76]. Two works aimed at methodology development were reviewed, one dealing with matrix effects [12] and the second comparing different extraction approaches [13]. Several other particles have been studied in biological matrices [44].

In summary, single‐particle ICP‐MS has proven to be a powerful and adaptable tool for the analysis of a wide variety of metal‐containing NPs across diverse matrices. Although the field has traditionally focused on well‐characterized and readily available particles such as Ag, Au, and TiO_2_, recent research has begun to explore more complex and environmentally relevant scenarios. Studies investigating in situ particle behavior, bioaccumulation, and release due to anthropogenic or medical sources are gradually increasing, reflecting a shift toward more applied and realistic assessments of NP exposure. Additionally, emerging materials such as Pt, CuO, and CeO_2_ are gaining attention due to their growing use and potential health risks, further expanding the analytical scope of spICP‐MS. As concerns about nanomaterial safety rise, the continued development of reliable and efficient analytical methodologies remains critical to advancing our understanding of NP fate, behavior, and potential impact in biological and environmental systems. This needs to be supported in the future by multidisciplinary research understanding of the effect of particles of different chemical compositions, sizes, and concentrations on the living organism. These new tools should be offered to toxicologists and medical and environmental researchers to find out more about the actual impact of anthropogenic particles on our environment.

### 2.3 spICP‐MS Technique Limitations and Considerations

Although the spICP‐MS technique is well established with a huge potential for the standardized methodology, it still faces a few limitations such as the assumption that all analyzed particles are perfectly spherical, the analysis of only one element (outside of TOF instruments) even though the particles may have multielemental/oxide composition, different approaches to calculating the transport efficiency, continual background of free ions as well as matrix‐derived interferences, lack of suitable reference matrix‐matched materials, and so forth [9].

#### 2.3.1 Estimation of Small NPs

One of the greatest advantages of spICP‐MS seems to be the ability to simultaneously measure the ionic and particle forms of an element [77]. However, in real samples where the ionic content of an element might be higher, this can become a double‐edged sword that significantly limits the technique's ability to detect smaller particles (roughly <10 nm, please see Table 1). This often manifests itself as a relatively high limit for the smallest detectable particle, which may result in incomplete distribution diagrams. The other negative side of this is that the size of the smallest detectable particle is highly dependent on the measured element and isotope, respectively. This is illustrated in Table 1, where sizes for the smallest detectable particle for individual elements found through the reviewing process are presented. Despite silver particles being ideal candidates for method validation due to their negligible natural background, the size of the smallest detectable particle below 10 nm has not been reported (Table 1). This may, in part, be caused by the low sensitivity of modern analytical instruments. For more naturally occurring elements like Ti or Si, sizes for the smallest detectable particle climbed up to 48.6 or 350 nm, respectively. These examples are, of course, extremes found in the reviewed studies. However, it illustrates the issue well. The minimal detectable size is dependent on the sensitivity of the instrument for the given elements and isotopes, respectively. Where Ag and Au are relatively “interference‐free” elements, something like Si particles could be hard to detect because a lot of the instrumental parts are made out of silica glass, plus the most abundant isotope ^28^Si is heavily interfered by ^14^N_2_. This issue is usually dealt with by an additional dilution of the sample to lower the concentration of ionic forms. However, this also has its own risk in lowering the number of particles entering the instrument. A novel approach has been deployed by Fréchette‐Viens et al. [78] where a Chelex‐100 ion‐exchange column separating free metal cations and NPs fraction was directly coupled to ICP‐MS throughout the single‐particle analysis, leading to more accurate determination of size distribution, lower size detection limits, and up to 95% reduction in background signal. To date, this promising approach is not widely used, especially not in the analysis of biological matrices. More work needs to be done to see if this approach could become a routine addition to spICP‐MS analysis.

All of this, however, is a part of an even bigger issue that is spICP‐MS data treatment. To distinguish a particle signal from the ionic background, a statistical calculation is necessary to establish the so‐called threshold. This threshold is a limit that differentiates between a background signal and a signal that is attributed to a particle being introduced into the instrument. How this calculation should be carried out is not agreed upon, and it is highly dependent on the calculating instrument used. Every manufacturer offers their own software. However, how the software does its threshold calculations is a manufacturer's secret. This prompted researchers to do the processing on their own, either by utilizing common software like Origin or Excel or by making their own software in programming languages like MATLAB and Python. All of the available data processing options have been recently summarized in a study by Chronakis [79]. Even when we take user‐friendly open‐source software like SPCal, which offers easy and quick single‐particle data processing, the calculation variables usually come down to the researcher's experience [80]. Furthermore, there is the issue of the real composition of the analyzed particles. In single‐particle size calculations, it is necessary to know the density of the particle. This is easy when we consider reference materials bought from manufacturers with a certificate. However, when we consider the naturally occurring particles, their exact composition is pure guesswork, which again comes down to researchers’ preferences and experience. The abovementioned issues with single‐particle data processing are the biggest hindrance to establishing spICP‐MS as a golden technique for particle analysis and limit its use to purely qualitative analysis with the additional bonus of rough estimation of particle size and concentration, which are both heavily affected by the researcher's input. This must be held in mind when publishing, and every calculation input should be properly reported. Despite these challenges, spICP‐MS remains a uniquely powerful technique due to its high sensitivity, single‐particle resolution, ability to provide quick rough particle size and number estimations, and work with complex matrices. These are advantages that strongly justify continued efforts to overcome current limitations and advance the method further. There are already ways to overcome some of the shortcomings. For example, the newly developed technique TDA‐ICP‐MS, where metal cations and NPs form could provide well‐distinguished peaks in the Taylogram with certain Dh_1_ and Dh_2_ values (hydrodynamic sizes), thus allowing for the detection of NPs without practically any limitation [81]. Moreover, the peak corresponding to the low ionic form fraction can be completely covered by the NPs signal, making a TDA‐ICP‐MS unique for tracking NPs and their mixtures in the complex alkaline/enzymatic extracts, buffered particles suspensions, or even directly in the body fluids [81, 82].

#### 2.3.2 Lack of Matrix‐Matched Certified Reference Materials

Another issue is connected with the sample preparation and the lack of any commercially available matrix‐matched reference materials. Thus, making it hard to validate these extraction methods and generally the whole ICP‐MS‐based approaches, the way around is using experiments by spiking widely available particle reference materials like Au and Ag into the sample matrix. This, of course, has its own shortcomings. Mainly, it does not reflect how effectively particles in the matrix are freed by the extraction process. These types of validation are harder to do for particle count than for size. On the market, there are only a few reference materials that come with a particle concentration in their certificate, and mostly they report this number as “informative” or “indicative.” There was a recent breakthrough in this regard with reference material LGCQC5050, which is the first commercially available NP reference material with a particle number concentration that is SI‐traceable. This helped spICP‐MS to mature enough to get its own ISO standardization (ISO/TS 19590:2024) that outlines how to perform accurate and reproducible spICP‐MS analysis in regard to: sample preparation; instrument tuning; calibration of transport efficiency; data acquisition and processing. Although this material has not been fully certified, it offers an invaluable tool for spICP‐MS quality control. Although spICP‐MS has progressed significantly and is now supported by emerging ISO standardization, such as ISO/TS 19590:2024, these advancements primarily address the analytical method itself rather than its application to complex real‐world biological samples. In such matrices, many of the issues discussed (background interference, matrix effects, lack of reference materials, and variability in data treatment) still persist and limit the technique's full potential. Nonetheless, these limitations do not undermine the value of the method, provided that researchers transparently report all experimental parameters, data processing choices, and assumptions. This transparency is crucial for reproducibility and comparability across studies. Looking ahead, other hyphenated ICP‐MS may offer complementary solutions to some of these persistent challenges, especially in distinguishing ionic and particulate species without affecting the reliability of results. However, spICP‐MS remains a uniquely powerful tool with unmatched speed, sensitivity, and particle‐level resolution. With continued development in areas such as data standardization, reference materials, and extraction protocols, it has the potential to evolve into a truly robust and quantitative technique for NP analysis in even the most challenging sample types.

### 2.4 spLA‐ICP‐MS

One of the newly arising single‐particle techniques is particle detection with laser ablation spICP‐MS. This technique, similar to classic spICP‐MS, bypasses the sample preparation issue by directly sampling sample surface. Unlike solution‐based spICP‐MS methods, spLA‐ICP‐MS preserves the spatial distribution of NPs within biological matrices, providing critical insights into their localization, transport, and potential biological interactions. By eliminating matrix decomposition/dissolution steps, this technique minimizes contamination, small particle dissolution, and size distribution alteration risks. The ability to perform high‐resolution elemental mapping further strengthens its role in studying NP fate in tissues, cells, and other biological specimens, making it a potentially strong tool for nanotoxicology, bioimaging, and biomedical research [83]. Despite its promising abilities, the use of this technique for NPs tracking in biological samples is still very limited with only four studies published since 2020 (Table 2). So far, spLA‐ICP‐MS has been applied primarily to investigate NP distribution, translocation, and degradation directly within biological tissues [84, 85]. Studies have demonstrated its ability to detect intact particles and distinguish them from their ionic degradation products, enabling detailed toxicokinetic assessments in organs such as the spleen, liver, kidney, and lungs [85]. A common strategy for quantitative analysis involves the use of matrix‐matched gelatine standards spiked with NPs [60, 84, 85], which help account for matrix effects and allow for particle size and concentration estimation. This approach remains the most prevalent calibration method, often supplemented by comparisons with other techniques such as spICP‐MS or TEM to validate size distributions. In terms of instrumentation, infrared (IR) lasers have shown significant advantages over ultraviolet (UV) systems by reducing particle fragmentation and nonspecific desorption, resulting in higher particle detection efficiency and better spatial resolution [86]. This may point at the fact that the common UV laser ablation unit commonly deployed in classic LA‐ICP‐MS analysis of solid samples might not be suitable for spLA‐ICP‐MS experiments. The pioneering studies highlight the technique's ability to provide high‐resolution spatial and size‐resolved information while maintaining NP integrity. These findings demonstrate its potential for unequivocal tracking NP fate in living organisms. However, several challenges must be addressed to broaden its applicability. Future research should focus on enhancing calibration strategies, developing better matrix‐matched standards, and optimizing laser ablation parameters to improve detection efficiency. Expanding the range of NPs analyzed, incorporating long‐term exposure studies, and refining data processing methods will be essential steps to fully unlock the potential of spLA‐ICP‐MS for advanced bioimaging and nanotoxicological assessments.

**TABLE 2:** Results of the literature search for studies where spLA‐inductively coupled plasma mass spectrometry (ICP‐MS) was utilized in the analysis of biological matrices.

Analyzed particles (size)	Sample matrix	Laser systems	MS instrument manufacturer	Mass analyzer	Ref.
Au (20 nm)	Spheroid prepared from human carcinoma cells	IR ablation system: OPOTEK Opolette 2940: 2940 nm flashlamp‐based pump laser UV ablation system: Teledyne Photon Machines LSX‐213 G2+: 213 nm Nd:YAG laser	Agilent Technologies	Q	[86]
Au (10, 30, and 50 nm)	Mouse spleen	UV ablation system: Teledyne Photon Machines LSX‐213 G2+: 213 nm Nd:YAG laser	Thermo Fisher	QQQ	[84]
Ag (50, 60, and 80 nm)	Mouse spleen, liver and kidney	UV ablation system: Electro Scientific Industries NWR213: 213 nm Nd:YAG laser	Perkin Elmer	Q	[85]
CeO_2_	Rat spleen	UV ablation system: Teledyne Photon Machines NWR193: 193 nm ArF excimer	Agilent Technologies	QQQ	[60]

*Note*: It contains information on the type and size of analyzed particles, sample matrix, type of laser system, MS instrument manufacturer, and type of mass analyzer used.

Abbreviations: Q = quadrupole; QQQ = triple quadrupole; SF = sector‐field; TOF = time‐of‐flight.

## 3 Hyphenated ICP‐MS Techniques for NP Analysis

In comparison with a spICP‐MS, the hyphenation of ICP‐MS with separation techniques allows the simultaneous analysis of NP mixtures with different particle types varying in sizes, as well as the determination of advanced characteristics such as aggregation or agglomeration status, and concentration of ionic form (dissolution study) [87]. The field‐flow fractionation, particularly asymmetric flow FFF for the rest of the section for clarity, and HPLC operated in hydrodynamic chromatography and SEC modes have already been utilized. As it was explained in Section 1, AF4 is the only mode used in hyphenation to ICP‐MS for NP detection. However, using the term FFF‐ICP‐MS yielded more results when doing the literature search.

AF4 separates particles based on their diffusion coefficients in a laminar cross‐flow field within a thin channel, enabling high‐resolution fractionation across a wide size range, including larger NPs and macromolecules [88]. SEC and HDC, on the other hand, rely on differences in the size of analytes as they pass through porous or packed columns. SEC separates molecules based on their ability to enter the pores of the stationary phase [89], whereas HDC uses packed columns where smaller particles are retained longer due to restricted access to faster flow paths (center of the flow) in laminar flow that are occupied by larger particles [5]. Although all three methods are effective for size‐based separations, AF4 offers higher versatility for polydisperse or fragile samples, whereas SEC and HDC are generally simpler and more accessible. However, their application in real biological matrices remains limited. One contributing factor is the relatively low recognition and availability of HDC in the analytical community [5]. Moreover, both SEC and AF4 have traditionally been more widely used for the separation of large biomolecules or polymers, rather than for detailed NP analysis in complex biological environments [90]. It is obvious from the literature search (Table 3) that these techniques are not widely deployed in NP detection in biological matrices. Only three relevant research studies have been found for FFF‐ICP‐MS, and two for each SEC‐ICP‐MS and HDC‐ICP‐MS. FFF coupled to ICP‐MS has been applied to size‐resolved analysis of NPs in matrices such as human urine, blood, and serum [42], animal feces [56], and skin receptor fluids [91]. It has shown effective separation of NPs down to a few nanometers in diameter (down to 2 nm). However, FFF‐ICP‐MS is limited by relatively high detection limits due to sample dilution during separation, the need for precise flow control, and longer analysis times, which increase operational complexity and cost. The SEC‐ICP‐MS has proven useful in studying NP behavior in simulated physiological fluids, particularly in monitoring metal ion release from NPs as a function of pH and other matrix conditions. Its ability to differentiate Cd^2+^ from intact quantum dots enables insights into NP dissolution, transformation, and potential toxicity [92]. SEC‐ICP‐MS is also used for the identification of naturally occurring metal‐based NPs in biological tissues [26]. HDC‐ICP‐MS (using PL‐PSDA Type 1 column with a nominal separation range of 5–300 nm, a length of 80 cm, and an internal diameter of 7.5 mm) has found its use in distinguishing between ionic and particulate Ag in animal feces [56, 93]. It appears that the technique is significantly underutilized, likely due to a lack of awareness, given that both studies were conducted by the same research group.

**TABLE 3:** Summary of studies employing hyphenated inductively coupled plasma mass spectrometry (ICP‐MS) techniques for the analysis of nanoparticles in biological matrices.

Analyzed particles (size)	Sample matrix	Hyphenated technique	System description	MS instrument manufacturer	Mass analyzer	Ref.
Gd‐containing polysiloxane NPs (5.5 nm)	Urine and serum	CE TDA	Agilent Technologies 7100 capillary electrophoresis system—70 cm × 75 µm thermally coated capillary with hydroxypropylcellulose	Perkin Elmer	Q	[61]
Fe_3_O_4_@COOH, Fe_3_O_4_, Fe_3_O_4_@Au, Fe_3_O_4_@PEG, Fe_3_O_4_@PEI, Fe_3_O_4_@Citr	Albumin and transferrin in phosphate buffer with NaCl	CE	Agilent Technologies 7100 capillary electrophoresis system—polyimide‐coated fused silica capillary, 70 cm × 75 µm	Agilent Technologies	QQQ	[98]
Fe_2_O_3_@SiO_2_ COOH NPs (60 nm)	Buffer H_3_PO_4_/NaOH, pH 7.4, containing polymyxin B	CE	Agilent Technologies 7100 capillary electrophoresis system—fused silica capillary, 47 cm × 50 µm	Agilent Technologies	Q	[99]
Fe SPIONs (15.3 and 20.2 nm)	Buffer H_3_PO_4_/NaOH, pH 7.4 containing 100 mM NaCl and HSA	CE	Agilent Technologies 7100 capillary electrophoresis system—CE polyimide‐coated fused silica capillary, 70 cm × 75 µm	Agilent Technologies	QQQ	[100]
TiO_2_ (5 and 60 nm)	Lip balm and toothpaste	CE	Agilent Technologies G1600AX capillary electrophoresis system, fused silica capillary, 65 cm × 75 µm	Agilent Technologies	Q	[102]
Au‐cisPt (10 nm)	Buffer HEPES 7.4, tricine 8.0	CE	Agilent Technologies 7100 capillary electrophoresis system—fused silica capillary, 70 cm × 75 µm	Agilent Technologies	QQQ	[101]
Au@SiO*_x_*	Serum, buffer H_3_PO_4_/NaOH pH 7.4 containing BSA and/or FET	TDA	Agilent Technologies 7100 capillary electrophoresis system—HPC capillary, 70 cm × 50 µm	Perkin Elmer	Q	[106]
Ga‐containing theranostic nanoparticle (2.3 nm)	Urine, cerebrospinal fluid, and undiluted serum, phosphate buffer 7.4, Tris NaCl	TDA	Agilent Technologies 7100 capillary electrophoresis system—HPC capillary, 70 cm × 75 µm	Perkin Elmer	Q	[82]
CdSe@ZnS (4.6 nm)	Simulated human body fluids: Gastric fluid, sweat, Gamble's solution and artificial lysosomal fluid	SEC‐ICP‐MS	Agilent Technologies 1200 LC system—SEC column: 500 Å pore size, 250 × 4.6 mm; mobile phase: 0.1 mM EDTA, 0.2 wt% SDS	Agilent Technologies	QQQ	[92]
HgSe (<40 nm)	Cetacean liver and muscles	SEC‐ICP‐MS	Agilent Technologies 1200 LC system—Amino column 1000 Å pore size, 250 × 4.6 mm, mobile phase: 2% FL‐70 (surfactant, Fisher Scientific) and 2 mM Na_2_S_2_O_3_	Agilent Technologies	QQQ	[26]
Ag (20 and 40 nm)	Pig and chicken simulated intestinal fluid	HDC‐ICP‐MS	Waters 2796 Bioseparations module—PLPSDA Type 1 column, 800 × 7.5 mm	Perkin Elmer	Q	[93]
Ag (10, 20, 40, and 60 nm)	Pig and chicken feces	HDC‐ICP‐MS	Waters 2796 Bioseparations module—PLPSDA Type 1 column, 800 × 7.5 mm	Perkin Elmer	Q	[56]
Ag (20, 60, and 100 nm) Au (5, 20, 40, and 60 nm)	Human urine, blood, serum	FFF‐ICP‐MS	Eclipse Dualtec FFF system (Wyatt Technology)—153 mm flow cell, 10 kDa regenerated cellulose membrane, 350 µm spacer	Thermo Fisher	Q	[42]
Ag (10, 20, 40, and 60 nm)	Pig and chicken feces	FFF‐ICP‐MS	AF2000 FFF system (Postnova)—140 × 20 mm trapezoidal channel, 5 kDa polyether sulfonate membrane, 350 µm spacer	Perkin Elmer	Q	[56]
Synthetic amorphous silica (SAS)	Receptor fluid after skin penetration test (phosphate buffer saline PBS)	FFF‐ICP‐MS	AF2000 FFF system (Postnova)—10 kDa regenerated cellulose membrane, 350 µm channel	Agilent Technologies	Q	[91]

*Note*: The table includes information on the type and size of the analyzed particles, sample matrix, separation technique used, system configuration, MS instrument manufacturer, and type of mass analyzer applied.

Abbreviations: CE = capillary electrophoresis; FFF = field‐flow fractionation; HDC = hydrodynamic chromatography; Q = quadrupole; QQQ = triple quadrupole; SEC = size‐exclusion chromatography; TDA = Taylor dispersion analysis.

On top of that, the capillary electrophoresis (CE) and Taylor dispersion techniques started to be extensively employed for NP characterization in the past two decades, as they can rapidly provide valuable information in terms of NPs’ hydrodynamic size, size distribution, and surface characteristics, such as surface charge density, zeta potential, and interaction with biomolecules and other biological systems (changes solvation layers, binding parameters) even for NP mixtures with minimal or no sample preparation step [94, 95, 96]. Considering that changes of abovementioned properties are closely related to the interaction of NPs with other components, typically proteins in biological samples, and/or NPs of other types if present together in the sample matrix [81], and the fact that CE takes place in free solution, it is obvious that CE offers a powerful tool that enables the study of interactions between NPs and other present species [81]. These other constituents are naturally occurring along with the NP analyte in real samples or can be simply added to the sample and/or running buffer to simulate real‐life conditions. Nowadays, CE is being more and more often hyphenated to ICP‐MS, which brings benefits, such as lower detection limits, wider linear dynamic range, and element‐specific detection, which is particularly advantageous in the case of complex samples where biological samples undoubtedly belong [97]. Interestingly, despite indisputable advantages, there are only six studies dealing with NP analysis in biological‐like media in the monitored period (2020–present) via CE‐ICP‐MS (Table 3). Only one study deals with the analysis of NPs in real biological samples (urine and serum matrix) [61], whereas four works are focused on NPs’ analysis in mimicked biological matrix (addition of biological constituent, especially protein(s), into running buffer and thus study of interactions with protein ligands) [98–101], and one is dedicated to NPs’ analysis in personal care products (lip balm and toothpaste) [102]. Here, the most investigated are variously modified iron‐based NPs. All the reviewed studies employed Agilent CE systems, suggesting a strong link between the instrumentation choice and the availability of an integrated Agilent CE‑ICP‑MS hyphenation kit. The utilization of the technique shows its capability for studying NP interactions with biomolecules, assessing drug delivery systems, and screening NP presence and behavior.

TDA has gained considerable attention for the accurate and reliable determination of hydrodynamic diameter from sub nm to µm, which covers the NP range. Unlike the size of the internal core in the “dry state,” the hydrodynamic diameter is a crucial parameter reflecting the real size of the solvated particle in suspension, taking into account surface modifications (coatings) as well as dynamic changes onto NP in biological media (e.g., protein corona) playing a key role, for instance, in cellular uptake [103]. TDA is an absolute method, consuming only pico‐nanoliters of sample volume, based on the determination of diffusion coefficient via the Taylor‐Aris equation, which can be turned into hydrodynamic radius using the Stokes–Einstein formula. Moreover, TDA can be conducted on a common CE instrument, because it offers all needed functions and parts such as an injection device, capillary, and pump [6, 104, 105]. Similarly to CE, TDA can be easily connected to an ICP‐MS, which is particularly advantageous in the case of complex biological samples, where the signal of interest can be overlapped by impurities or if NPs of various materials are present [81, 82]. TDA‐ICP‐MS opens a new window for the ICP‐MS laboratories with older instruments incapable of catching the fast transient signals created by single particles. The Gaussian peak duration lasts for more than tens of seconds even for ultrasmall NPs with a nanometer size [81], which brings new opportunities for multielemental (multi‐NP screening) and isotope ratio studies, especially for the isotopically enriched NPs [81], and accurate quantitative analyses without any need for the NPs certified reference materials due to the structure‐independent detection of ICP‐MS. Even though TDA has been a marginal matter for a long time, its usage is nowadays rapidly increasing because of many benefits as discussed by Gouyon et al. [6]. As TDA‐ICP‐MS is a newly emerging technique, its usage in biological samples has been limited (Table 3). The sample matrix was either bodily fluids [61, 82] or a buffer simulating a biological environment [106]. Nevertheless, these first studies have shown the great potential of the technique to detect particles with sizes down to 2 nm and even monitor such particles in media like human blood serum or urine [82]. From the relatively small number of published studies and limited application on real biological samples for both CE‐ICP‐MS and TDA‐ICP‐MS, it is clear that these techniques are still in their early stages of development, and more work needs to be done to assess their real value in NP analysis in biological samples. However, the potential they show seems promising.

## 4 Conclusion

Over the past decades, the demand for the development of sensitive and robust analytical approaches for the detection and advanced characterization of NPs has grown rapidly. The review describes the current state of the art in the ICP‐MS‐based detection of NPs in animal, human tissues, and biological fluids. The literature search focused on two different groups of techniques: (1) single‐particle techniques and (2) separation techniques hyphenated to ICP‐MS. The review was focused on the widely adopted spICP‐MS applications, which allow the determination of NPs size and size distribution in the prepared extracts. Attention was also paid to sample preparation protocols, where the alkaline, enzymatic, acidic, and dilution methods were discussed and compared. The trend seems to be shifting more toward alkaline digestion with its simplicity and more reliable matrix digestion. A unique case of sample digestion comes from acidic digestion protocols, offering fast and high‐throughput methodologies for the extraction of chemically persistent NPs like TiO_2_. The distribution of sample types highlights a strong emphasis on accessible and ethically feasible matrices for assessing NP behavior, while also underscoring the ongoing challenges and limitations associated with studying internal tissues, particularly in humans. A recent breakthrough with the first commercially available NP reference material LFCQC5050, with SI‐traceable particle number concentration, has led to spICP‐MS acquiring its own ISO standardization (ISO/TS 19590:2024). This has been a significant step for the analytical technique. The future of spICP‐MS is increasingly oriented toward TOF‐based instruments due to their capability for true multielement detection, whereas the development of new researcher‐made software is expected to further advance the field by offering dedicated spICP‐TOF‐MS data processing with greater transparency and flexibility than manufacturer‐provided solutions. Single‐particle LA‐ICP‐MS has slowly emerged as a new technique overcoming sample preparation issues connected with spICP‐MS. It preserves the spatial distribution of NPs within biological matrices, providing better insight into the NP transport and potential biological interactions. Despite the promising capabilities of single‐particle LA‐ICP‐MS without minimal sample preparation, its application in biological matrices remains underexplored.

Although spICP‐MS remains a cornerstone technique for NP analysis due to its sensitivity and ability to provide quick single‐particle size and concentration data, its limitations in resolving NP mixtures, differentiating dissolved from particulate forms, and characterizing interactions within complex biological matrices emphasize the need for complementary analytical approaches. Hyphenated methods such as FFF‐ICP‐MS, SEC‐ICP‐MS, and HDC‐ICP‐MS have demonstrated their potential to address these gaps by enabling size‐based separation and detailed insights into aggregation, agglomeration, and dissolution behavior in complex matrices such as urine, blood, serum, and feces. Although these methods provide valuable information, their utilization in scientific literature is rare and, as such, points to a potential lack of awareness of these methods.

Furthermore, CE‐ICP‐MS has emerged as a versatile method for probing surface charge, size distribution, and NP–biomolecule interactions, with recent applications focusing on mimicked biological matrices and only one study conducted on real human samples. The reviewed literature shows CE‐ICP‐MS being used primarily with Agilent instrumentation, suggesting that hardware availability may currently limit broader adoption. Likewise, TDA‐ICP‐MS, though still in its infancy, has demonstrated the capacity to detect particles as small as 2 nm in challenging matrices like human serum and urine and offers a path forward for ICP‐MS labs lacking the time‐resolution needed for traditional spICP‐MS. Its unique ability to produce broad, quantifiable peaks over several seconds enables multielement screening and isotope ratio analysis, which are difficult to perform with traditional single‐particle methods.

Taken together, these findings demonstrate that the future of ICP‐MS‐based analysis of NPs in biological systems lies not in one dominant technique but in the integration of complementary methods. Although spICP‐MS, particularly when coupled with ICP‐TOF‐MS, offers powerful single‐particle, multielement detection, its broader adoption is limited by the scarcity and high cost of TOF instruments, which continues to hinder progress in this area. Separation and dispersion‐based techniques enrich the analytical picture by enabling speciation, interaction studies, and realistic simulation of physiological conditions. As new open‐source and researcher‐developed software improves the transparency and accessibility of spICP‐TOF‐MS data processing, and as CE‐ and TDA‐based methods become more mature, the harmonized use of these tools will be key to advancing our understanding of NP fate, behavior, and safety in biological systems.

## Author Contributions

**Filip Gregar**: writing – original draft, conceptualization, visualization. **Daniel Baron**: writing – original draft, conceptualization. **Tomáš Pluháček**: writing – review and editing, conceptualization, supervision, resources.

## Conflicts of Interest

The authors declare no conflicts of interest.

## Declaration of Use of AI‐Assisted Technologies

In the development of this work, ChatGPT was utilized to enhance the readability of a few parts of the text. Following the utilization of this tool/service, the author(s) reviewed and edited the material as necessary and take(s) full responsibility for the content of the publication.

## References

[1] H. Nasrollahpour , B. J. Sánchez , M. Sillanpää , and R. Moradi , “Metal Nanoclusters in Point‐of‐Care Sensing and Biosensing Applications,” ACS Applied Nano Materials 6, no. 14 (2023): 283–292.

[2] A. F. Burlec , A. Corciova , M. Boev , et al., “Current Overview of Metal Nanoparticles' Synthesis, Characterization, and Biomedical Applications, With a Focus on Silver and Gold Nanoparticles,” Pharmaceuticals 16, no. 10 (2023): 1410. 37895881 10.3390/ph16101410 PMC10610223

[3] T. M. Joseph , D. K. Mahapatra , A. Esmaeili , et al., “Nanoparticles: Taking a Unique Position in Medicine,” Nanomaterials 13, no. 3 (2023): 574. 36770535 10.3390/nano13030574 PMC9920911

[4] S. Mourdikoudis , R. M. Pallares , and N. T. K. Thanh , “Characterization Techniques for Nanoparticles: Comparison and Complementarity Upon Studying Nanoparticle Properties,” Nanoscale 10, no. 27 (2018): 12871–12934. 29926865 10.1039/c8nr02278j

[5] A. K. Brewer , “Hydrodynamic Chromatography: The Underutilized Size‐Based Separation Technique,” Chromatographia 84, no. 9 (2021): 807–811.

[6] J. Gouyon , A. Boudier , F. Barakat , A. Pallotta , and I. Clarot , “Taylor Dispersion Analysis of Metallic‐Based Nanoparticles—A Short Review,” Electrophoresis 43, no. 23–24 (2022): 2377–2391. 36153831 10.1002/elps.202200184

[7] B. Meermann and V. Nischwitz , “ICP‐MS for the Analysis at the Nanoscale—A Tutorial Review,” Journal of Analytical Atomic Spectrometry 33, no. 9 (2018): 1432–1468.

[8] E. Bolea , M. S. Jimenez , J. Perez‐Arantegui , et al., “Analytical Applications of Single Particle Inductively Coupled Plasma Mass Spectrometry: A Comprehensive and Critical Review,” Analytical Methods 13, no. 25 (2021): 2742–2795. 34159952 10.1039/d1ay00761k

[9] A. Laycock , N. J. Clark , R. Clough , R. Smith , and R. D. Handy , “Determination of Metallic Nanoparticles in Biological Samples by Single Particle ICP‐MS: A Systematic Review From Sample Collection to Analysis,” Environmental Science: Nano 9, no. 2 (2022): 420–453. 35309016 10.1039/d1en00680k PMC8852815

[10] S. Naasz , S. Weigel , O. Borovinskaya , et al., “Multi‐Element Analysis of Single Nanoparticles by ICP‐MS Using Quadrupole and Time‐of‐Flight Technologies,” Journal of Analytical Atomic Spectrometry 33, no. 5 (2018): 835–845.

[11] A. Chalifoux , M. Hadioui , N. Amiri , and K. J. Wilkinson , “Analysis of Silver Nanoparticles in Ground Beef by Single Particle Inductively Coupled Plasma Mass Spectrometry (SP‐ICP‐MS),” Molecules (Basel, Switzerland) 28, no. 11 (2023): 4442. 37298916 10.3390/molecules28114442 PMC10254385

[12] Y. Y. Huang , J. T. S. Lum , and K. S. Y. Leung , “Single Particle ICP‐MS Combined With Internal Standardization for Accurate Characterization of Polydisperse Nanoparticles in Complex Matrices,” Journal of Analytical Atomic Spectrometry 35, no. 10 (2020): 2148–2155.

[13] Y. Y. Huang , J. T. S. Lum , and K. S. Y. Leung , “An Integrated ICP‐MS‐Based Analytical Approach to Fractionate and Characterize Ionic and Nanoparticulate Ce Species,” Analytical and Bioanalytical Chemistry 414, no. 11 (2022): 3397–3410. 35129641 10.1007/s00216-022-03958-z

[14] S. T. Lancaster , G. Peniche , A. Alzahrani , et al., “Mercury Speciation in Scottish Raptors Reveals High Proportions of Inorganic Mercury in Scottish Golden Eagles (*Aquila chrysaetos*): Potential Occurrence of Mercury Selenide Nanoparticles,” Science of the Total Environment 829 (2022): 154557. 35302012 10.1016/j.scitotenv.2022.154557

[15] L. Paton , T. T. Moro , T. Lockwood , et al., “AF_4_‐MALS‐SP ICP‐ToF‐MS Analysis Gives Insight Into Nature of HgSe Nanoparticles Formed by Cetaceans,” Environmental Science: Nano 11, no. 5 (2024): 1883–1890.

[16] K. El Hanafi , B. Gomez‐Gomez , Z. Pedrero , P. Bustamante , Y. Cherel , and D. Amouroux , “Simple and Rapid Formic Acid Sample Treatment for the Isolation of HgSe Nanoparticles From Animal Tissues,” Analytica Chimica Acta 1250 (2023): 340952. 36898809 10.1016/j.aca.2023.340952

[17] J. Toledano‐Serrabona , D. P. de Moraes , S. González‐Morales , et al., “Tracking Soluble and Nanoparticulated Titanium Released *In Vivo* From Metal Dental Implant Debris Using (Single‐Particle)‐ICP‐MS,” Journal of Trace Elements in Medicine and Biology 77 (2023): 127143. 36871433 10.1016/j.jtemb.2023.127143

[18] F. Aureli , M. Ciprotti , M. D'Amato , et al., “Determination of Total Silicon and SiO_2_ Particles Using an ICP‐MS Based Analytical Platform for Toxicokinetic Studies of Synthetic Amorphous Silica,” Nanomaterials 10, no. 5 (2020): 888. 32384606 10.3390/nano10050888 PMC7279390

[19] N. Liu , Y. Li , L. H. Liu , et al., “Administration of Silver Nasal Spray Leads to Nanoparticle Accumulation in Rat Brain Tissues,” Environmental Science & Technology 56, no. 1 (2022): 403–413. 34923819 10.1021/acs.est.1c02532

[20] H. Tao , K. Nagano , I. Tasaki , et al., “Development and Evaluation of a System for the Semi‐Quantitative Determination of the Physical Properties of Skin After Exposure to Silver Nanoparticles,” Nanoscale Research Letters 15, no. 1 (2020): 187. 32990829 10.1186/s11671-020-03421-x PMC7524913

[21] Y. Gao , R. Y. Zhang , H. Z. Sun , et al., “High‐Efficiency Mechanically Assisted Alkaline Extraction of Nanoparticles From Biological Tissues for spICP‐MS Analysis,” Analytical and Bioanalytical Chemistry 414, no. 15 (2022): 4401–4408. 35175388 10.1007/s00216-022-03972-1

[22] H. Z. Sun , D. Han , Y. Gao , et al., “Particle‐Size‐Dependent Biological Distribution of Gold Nanoparticles After Interstitial Injection,” Materials Chemistry Frontiers 6, no. 18 (2022): 2760–2767.

[23] T. Yan , H. Z. Sun , Y. H. Shi , et al., “Thoracic Interstitial Injection of Drug‐Liposomes in Mice for Treating Atherosclerosis,” Nano Research 16, no. 4 (2023): 5311–5321.

[24] B. Sadeghalvad and E. P. Gray , “Evaluation of Alternative Bases to TMAH for Tissue Extraction of ENMs From Tissues Prior to spICP‐MS Analysis,” Environmental Science: Nano 11, no. 10 (2024): 4309–4320.

[25] W. C. Zhang , S. W. Huo , S. X. Deng , et al., “ *In Vivo* Exposure Pathways of Ambient Magnetite Nanoparticles Revealed by Machine Learning‐Aided Single‐Particle Mass Spectrometry,” Nano Letters 24, no. 31 (2024): 9535–9543. 38954740 10.1021/acs.nanolett.4c01937

[26] X. Ji , L. Yang , F. Wu , et al., “Identification of Mercury‐Containing Nanoparticles in the Liver and Muscle of Cetaceans,” Journal of Hazardous Materials 424 (2022): 127759. 34801316 10.1016/j.jhazmat.2021.127759

[27] J. Wu , J. Q. Sun , T. Bosker , M. G. Vijver , and W. Peijnenburg , “Toxicokinetics and Particle Number‐Based Trophic Transfer of a Metallic Nanoparticle Mixture in a Terrestrial Food Chain,” Environmental Science & Technology 57 (2023): 2792–2803. 36747472 10.1021/acs.est.2c07660

[28] S. Kuehr , J. Klehm , C. Stehr , M. Menzel , and C. Schlechtriem , “Unravelling the Uptake Pathway and Accumulation of Silver From Manufactured Silver Nanoparticles in the Freshwater Amphipod *Hyalella azteca* Using Correlative Microscopy,” NanoImpact 19 (2020): 100239.

[29] Y. Sun , Y. Yang , F. Y. Tou , et al., “Extraction and Quantification of Metal‐Containing Nanoparticles in Marine Shellfish Based on Single Particle Inductively Coupled Plasma‐Mass Spectrometry Technique,” Journal of Hazardous Materials 424 (2022): 127383. 34879574 10.1016/j.jhazmat.2021.127383

[30] A. Bruvold , S. Valdersnes , K. Loeschner , and A. M. Bienfait , “Validation of a Method for Surveillance of Nanoparticles in Mussels Using Single‐Particle Inductively Coupled Plasma‐Mass Spectrometry,” Journal of AOAC International 107 (2024): 608–616. 38507699 10.1093/jaoacint/qsae024 PMC11223760

[31] H. Y. Lu , Y. J. Wang , and W. C. Hou , “Bioaccumulation and Depuration of TiO_2_ Nanoparticles by Zebrafish Through Dietary Exposure: Size‐ and Number Concentration‐Resolved Analysis Using Single‐Particle ICP‐MS,” Journal of Hazardous Materials 426 (2022): 127801. 34863574 10.1016/j.jhazmat.2021.127801

[32] F. Gallocchio , A. Moressa , F. Pascoli , et al., “Effect of TiO_2_ Nanoparticle on Bioaccumulation of Ndl‐PCBs in Mediterranean Mussels (*Mitilus galloprovincialis*),” Animals 13, no. 7 (2023): 1208. 37048464 10.3390/ani13071208 PMC10093413

[33] M. V. Taboada‐López , B. H. Leal‐Martínez , R. Domínguez‐González , P. Bermejo‐Barrera , P. Taboada‐Antelo , and A. Moreda‐Piñeiro , “Caco‐2 *In Vitro* Model of Human Gastrointestinal Tract for Studying the Absorption of Titanium Dioxide and Silver Nanoparticles From Seafood,” Talanta 233 (2021): 122494. 34215112 10.1016/j.talanta.2021.122494

[34] N. J. Clark , R. Clough , D. Boyle , and R. D. Handy , “Quantification of Particulate Ag in Rainbow Trout Organs Following Dietary Exposure to Silver Nitrate, or Two Forms of Engineered Silver Nanoparticles,” Environmental Science: Nano 8, no. 6 (2021): 1642–1653.

[35] M. E. Johnson , J. Bennett , A. R. M. Bustos , et al., “Combining Secondary Ion Mass Spectrometry Image Depth Profiling and Single Particle Inductively Coupled Plasma Mass Spectrometry to Investigate the Uptake and Biodistribution of Gold Nanoparticles in *Caenorhabditis elegans* ,” Analytica Chimica Acta 1175 (2021): 338671. 34330435 10.1016/j.aca.2021.338671

[36] Q. F. Zhou , L. H. Liu , N. A. Liu , B. He , L. G. Hu , and L. N. Wang , “Determination and Characterization of Metal Nanoparticles in Clams and Oysters,” Ecotoxicology and Environmental Safety 198 (2020): 110670. 32344268 10.1016/j.ecoenv.2020.110670

[37] Q. Yu , Z. Y. Zhang , F. A. Monikh , et al., “Trophic Transfer of Cu Nanoparticles in a Simulated Aquatic Food Chain,” Ecotoxicology and Environmental Safety 242 (2022): 113920. 35905628 10.1016/j.ecoenv.2022.113920

[38] A. Grasso , M. Ferrante , A. Moreda‐Pineiro , et al., “Dietary Exposure of Zinc Oxide Nanoparticles (ZnO‐NPs) From Canned Seafood by Single Particle ICP‐MS: Balancing of Risks and Benefits for Human Health,” Ecotoxicology and Environmental Safety 231 (2022): 113217. 35077994 10.1016/j.ecoenv.2022.113217

[39] J. Kalman , M. Connolly , F. Abdolahpur‐Monikh , et al., “Bioaccumulation of CuO Nanomaterials in Rainbow Trout: Influence of Exposure Route and Particle Shape,” Chemosphere 310 (2023): 136894. 36265710 10.1016/j.chemosphere.2022.136894

[40] B. Bocca , B. Battistini , V. Leso , et al., “Occupational Exposure to Metal Engineered Nanoparticles: A Human Biomonitoring Pilot Study Involving Italian Nanomaterial Workers,” Toxics 11, no. 2 (2023): 120. 36850996 10.3390/toxics11020120 PMC9962841

[41] S. Fernández‐Trujillo , M. Jiménez‐Moreno , A. Ríos , and R. D. R. Martín‐Doimeadios , “A Simple Analytical Methodology for Platinum Nanoparticles Control in Complex Clinical Matrices via SP‐ICP‐MS,” Talanta 231 (2021): 122370. 33965035 10.1016/j.talanta.2021.122370

[42] B. Bocca , B. Battistini , and F. Petrucci , “Silver and Gold Nanoparticles Characterization by SP‐ICP‐MS and AF4‐FFF‐MALS‐UV‐ICP‐MS in Human Samples Used for Biomonitoring,” Talanta 220 (2020): 121404. 32928420 10.1016/j.talanta.2020.121404

[43] M. Cabré , G. Fernández , E. González , J. Abellà , and A. Verdaguer , “Single Particle ICP‐MS: A Tool for the Characterization of Gold Nanoparticles in Nanotheranostics Applications,” Journal of Analytical Atomic Spectrometry 39, no. 10 (2024): 2508–2513.

[44] B. Bocca , V. Leso , B. Battistini , et al., “Human Biomonitoring and Personal Air Monitoring. An Integrated Approach to Assess Exposure of Stainless‐Steel Welders to Metal‐Oxide Nanoparticles,” Environmental Research 216 (2023): 114736. 36343713 10.1016/j.envres.2022.114736

[45] M. Hernández‐Postigo , A. Sánchez‐Cachero , M. Jiménez‐Moreno , and R. C. R. Martín‐Doimeadios , “Determination of Size and Particle Number Concentration of Metallic Nanoparticles Using Isotope Dilution Analysis Combined With Single Particle ICP‐MS to Minimise Matrix Effects,” Microchimica Acta 192, no. 1 (2024): 28. 39710788 10.1007/s00604-024-06894-0

[46] S. Salou , C. M. Cirtiu , D. Larivière , and N. Fleury , “Assessment of Strategies for the Formation of Stable Suspensions of Titanium Dioxide Nanoparticles in Aqueous Media Suitable for the Analysis of Biological Fluids,” Analytical and Bioanalytical Chemistry 412, no. 7 (2020): 1469–1481. 32034456 10.1007/s00216-020-02412-2

[47] I. Abad‐Alvaro , D. Leite , D. Bartczak , et al., “An Insight Into the Determination of Size and Number Concentration of Silver Nanoparticles in Blood Using Single Particle ICP‐MS (spICP‐MS): Feasibility of Application to Samples Relevant to *In Vivo* Toxicology Studies,” Journal of Analytical Atomic Spectrometry 36, no. 6 (2021): 1180–1192.

[48] M. Ferrante , A. Grasso , G. Giuberti , et al., “Behaviour and Fate of Ag‐NPs, TiO_2_‐NPs and ZnO‐NPs in the Human Gastrointestinal Tract: Biopersistence Rate Evaluation,” Food and Chemical Toxicology 176 (2023): 113779. 37062331 10.1016/j.fct.2023.113779

[49] K. Mehrabi , M. Dengler , I. Nilsson , et al., “Detection of Magnetic Iron Nanoparticles by Single‐Particle ICP‐TOFMS: Case Study for a Magnetic Filtration Medical Device,” Analytical and Bioanalytical Chemistry 414, no. 23 (2022): 6743–6751. 35864268 10.1007/s00216-022-04234-w

[50] D. M. Peloquin , E. J. Baumann , and T. P. Luxton , “Multi‐Method Assessment of PVP‐Coated Silver Nanoparticles and Artificial Sweat Mixtures,” Chemosphere 249 (2020): 126173. 32065993 10.1016/j.chemosphere.2020.126173 PMC7449241

[51] M. Cosmi , N. Gonzalez‐Quiñonez , P. T. Díaz , et al., “Evaluation of Nanodebris Produced by *In Vitro* Degradation of Titanium‐Based Dental Implants in the Presence of Bacteria Using Single Particle and Single Cell Inductively Coupled Plasma Mass Spectrometry,” Journal of Analytical Atomic Spectrometry 36, no. 9 (2021): 2007–2016.

[52] Y. U. Hachenberger , D. Rosenkranz , C. Kromer , et al., “Nanomaterial Characterization in Complex Media‐Guidance and Application,” Nanomaterials 13, no. 5 (2023): 18. 10.3390/nano13050922 PMC10005142 36903800

[53] J. Y. Wu , F. Y. Tou , Y. Yang , et al., “Metal‐Containing Nanoparticles in Low‐Rank Coal‐Derived Fly Ash From China: Characterization and Implications Toward Human Lung Toxicity,” Environmental Science & Technology 55, no. 10 (2021): 6644–6654. 33969690 10.1021/acs.est.1c00434

[54] T. N. Akbaba , N. Ertas , and O. Alp , “Characterization of the Silver Species Released From Clothing by Single Particle‐Inductively Coupled Plasma‐Mass Spectrometry Using a Microsecond Dwell Time,” Analytical Letters 55, no. 4 (2022): 580–595.

[55] C. Y. Zhang , Q. Zhang , Y. C. Zhao , D. Q. Dong , and L. J. Huang , “Determination of Titanium (IV) Oxide Nanoparticles Released From Textiles by Single Particle—Inductively Coupled Plasma—Mass Spectrometry (SP‐ICP‐MS),” Analytical Letters 57, no. 1 (2024): 82–91.

[56] M. S. Jiménez , M. Bakir , K. Ben‐Jeddou , E. Bolea , J. Pérez‐Arantegui , and F. Laborda , “Comparative Study of Extraction Methods of Silver Species From Faeces of Animals Fed With Silver‐Based Nanomaterials,” Microchimica Acta 190, no. 6 (2023): 204. 37160774 10.1007/s00604-023-05777-0 PMC10169895

[57] Y. Z. Sun , N. Liu , Y. Y. Wang , et al., “Monitoring AuNP Dynamics in the Blood of a Single Mouse Using Single Particle Inductively Coupled Plasma Mass Spectrometry With an Ultralow‐Volume High‐Efficiency Introduction System,” Analytical Chemistry 92, no. 22 (2020): 14872–14877. 32972134 10.1021/acs.analchem.0c02285

[58] R. J. B. Peters , A. G. Oomen , G. van Bemmel , et al., “Silicon Dioxide and Titanium Dioxide Particles Found in Human Tissues,” Nanotoxicology 14, no. 3 (2020): 420–432. 31994971 10.1080/17435390.2020.1718232

[59] F. Gregar , J. Gallo , D. Milde , et al., “In Vivo Assessment of TiO_2_ Based Wear Nanoparticles in Periprosthetic Tissues,” Analytical and Bioanalytical Chemistry 416, no. 16 (2024): 3785–3796. 38724776 10.1007/s00216-024-05320-x PMC11180632

[60] S. B. Seiffert , M. Elinkmann , E. Niehaves , et al., “Calibration Strategy to Size and Localize Multi‐Shaped Nanoparticles in Tissue Sections Using LA‐spICP‐MS,” Analytical Chemistry 95, no. 15 (2023): 6383–6390. 37023260 10.1021/acs.analchem.3c00022

[61] L. Labied , P. Rocchi , T. Doussineau , et al., “Biodegradation of Metal‐Based Ultra‐Small Nanoparticles: A Combined Approach Using TDA‐ICP‐MS and CE‐ICP‐MS,” Analytica Chimica Acta 1185 (2021): 339081. 34711326 10.1016/j.aca.2021.339081

[62] A. Hamad , K. S. Khashan , and A. Hadi , “Silver Nanoparticles and Silver Ions as Potential Antibacterial Agents,” Journal of Inorganic and Organometallic Polymers and Materials 30, no. 12 (2020): 4811–4828.

[63] J. M. Radziwill‐Bienkowska , P. Talbot , J. B. J. Kamphuis , et al., “Toxicity of Food‐Grade TiO_2_ to Commensal Intestinal and Transient Food‐Borne Bacteria: New Insights Using Nano‐SIMS and Synchrotron UV Fluorescence Imaging,” Frontiers in Microbiology 9 (2018): 794. 29740421 10.3389/fmicb.2018.00794 PMC5928251

[64] Z. J. Chen , Y. Wang , L. Zhuo , et al., “Effect of Titanium Dioxide Nanoparticles on the Cardiovascular System After Oral Administration,” Toxicology Letters 239, no. 2 (2015): 123–130. 26387441 10.1016/j.toxlet.2015.09.013

[65] R. Cornu , A. Béduneau , and H. Martin , “Ingestion of Titanium Dioxide Nanoparticles: A Definite Health Risk for Consumers and Their Progeny,” Archives of Toxicology 96, no. 10 (2022): 2655–2686. 35895099 10.1007/s00204-022-03334-x

[66] Z. J. Chen , D. Zhou , Y. Wang , et al., “Combined Effect of Titanium Dioxide Nanoparticles and Glucose on the Cardiovascular System in Young Rats After Oral Administration,” Journal of Applied Toxicology 39, no. 4 (2019): 590–602. 30427543 10.1002/jat.3750

[67] S. Salou , D. Larivière , C. M. Cirtiu , and N. Fleury , “Quantification of Titanium Dioxide Nanoparticles in Human Urine by Single‐Particle ICP‐MS,” Analytical and Bioanalytical Chemistry 413, no. 1 (2021): 171–181. 33123763 10.1007/s00216-020-02989-8

[68] R. A. Lavoie , T. D. Jardine , M. M. Chumchal , K. A. Kidd , and L. M. Campbell , “Biomagnification of Mercury in Aquatic Food Webs: A Worldwide Meta‐Analysis,” Environmental Science & Technology 47, no. 23 (2013): 13385–13394. 24151937 10.1021/es403103t

[69] Z. Gajdosechova , A. Brownlow , N. T. Cottin , et al., “Possible Link Between Hg and Cd Accumulation in the Brain of Long‐Finned Pilot Whales (*Globicephala melas*),” Science of the Total Environment 545 (2016): 407–413. 26748005 10.1016/j.scitotenv.2015.12.082

[70] Z. Gajdosechova , Z. Mester , J. Feldmann , and E. M. Krupp , “The Role of Selenium in Mercury Toxicity–Current Analytical Techniques and Future Trends in Analysis of Selenium and Mercury Interactions in Biological Matrices,” TrAC Trends in Analytical Chemistry 104 (2018): 95–109.

[71] X. M. Ji , L. Yang , F. X. Wu , et al., “Identification of Mercury‐Containing Nanoparticles in the Liver and Muscle of Cetaceans,” Journal of Hazardous Materials 424 (2022): 127759. 34801316 10.1016/j.jhazmat.2021.127759

[72] M. Jeyaraj , S. Gurunathan , M. Qasim , M.‐H. Kang , and J.‐H. Kim , “A Comprehensive Review on the Synthesis, Characterization, and Biomedical Application of Platinum Nanoparticles,” Nanomaterials 9, no. 12 (2019): 1719. 31810256 10.3390/nano9121719 PMC6956027

[73] D. Pedone , M. Moglianetti , E. De Luca , G. Bardi , and P. P. Pompa , “Platinum Nanoparticles in Nanobiomedicine,” Chemical Society Reviews 46, no. 16 (2017): 4951–4975. 28696452 10.1039/c7cs00152e

[74] E. Czubacka and S. Czerczak , “Are Platinum Nanoparticles Safe to Human Health?,” Medycyna Pracy 70 (2019): 487–495. 31162484 10.13075/mp.5893.00847

[75] Z. Li , S. Chang , S. Khuje , and S. Ren , “Recent Advancement of Emerging Nano Copper‐Based Printable Flexible Hybrid Electronics,” ACS Nano 15, no. 4 (2021): 6211–6232. 33834763 10.1021/acsnano.1c02209

[76] F. Gómez‐Rivera , J. A. Field , D. Brown , and R. Sierra‐Alvarez , “Fate of Cerium Dioxide (CeO_2_) Nanoparticles in Municipal Wastewater During Activated Sludge Treatment,” Bioresource Technology 108 (2012): 300–304. 22265985 10.1016/j.biortech.2011.12.113

[77] H. Goenaga‐Infante and D. Bartczak , “Single Particle Inductively Coupled Plasma Mass Spectrometry (spICP‐MS),” in Characterization of Nanoparticles (Elsevier, 2020).

[78] L. Fréchette‐Viens , M. Hadioui , and K. J. Wilkinson , “Practical Limitations of Single Particle ICP‐MS in the Determination of Nanoparticle Size Distributions and Dissolution: Case of Rare Earth Oxides,” Talanta 163 (2017): 121–126. 27886760 10.1016/j.talanta.2016.10.093

[79] M. I. Chronakis , B. Meermann , and M. von der Au , “The Evolution of Data Treatment Tools in Single‐Particle and Single‐Cell ICP‐MS Analytics,” Analytical and Bioanalytical Chemistry 417, no. 1 (2025): 7–13. 39230750 10.1007/s00216-024-05513-4 PMC11695396

[80] T. E. Lockwood , L. Schlatt , and D. Clases , “SPCal—An Open Source, Easy‐to‐Use Processing Platform for ICP‐TOFMS‐Based Single Event Data,” Journal of Analytical Atomic Spectrometry 40, no. 1 (2025): 130–136.

[81] D. Baron , T. Pluhacek , and J. Petr , “Characterization of Nanoparticles in Mixtures by Taylor Dispersion Analysis Hyphenated to Inductively Coupled Plasma Mass Spectrometry,” Analytical Chemistry 96, no. 14 (2024): 5658–5663. 38529586 10.1021/acs.analchem.4c00586 PMC11007675

[82] L. Labied , P. Rocchi , T. Doussineau , et al., “Taylor Dispersion Analysis Coupled to Inductively Coupled Plasma‐Mass Spectrometry for Ultrasmall Nanoparticle Size Measurement: From Drug Product to Biological Media Studies,” Analytical Chemistry 93, no. 3 (2021): 1254–1259. 33372768 10.1021/acs.analchem.0c03988

[83] J. S. Becker , A. Matusch , and B. Wu , “Bioimaging Mass Spectrometry of Trace Elements—Recent Advance and Applications of LA‐ICP‐MS: A Review,” Analytica Chimica Acta 835 (2014): 1–18. 24952624 10.1016/j.aca.2014.04.048

[84] I. D. Nordhorn , D. Dietrich , C. Verlemann , et al., “Spatially and Size‐Resolved Analysis of Gold Nanoparticles in Rat Spleen After Intratracheal Instillation by Laser Ablation‐Inductively Coupled Plasma‐Mass Spectrometry,” Metallomics 13, no. 6 (2021): mfab028. 33979446 10.1093/mtomcs/mfab028

[85] M. Wang , L. N. Zheng , B. Wang , et al., “Laser Ablation‐Single Particle‐Inductively Coupled Plasma Mass Spectrometry as a Sensitive Tool for Bioimaging of Silver Nanoparticles *In Vivo* Degradation,” Chinese Chemical Letters 33, no. 7 (2022): 3484–3487.

[86] M. Stiborek , L. Jindrichová , S. Meliorisová , et al., “Infrared Laser Desorption of Intact Nanoparticles for Digital Tissue Imaging,” Analytical Chemistry 94, no. 51 (2022): 18114–18120. 36514811 10.1021/acs.analchem.2c05216

[87] S. Fernández‐Trujillo , M. Jiménez‐Moreno , N. Rodríguez‐Fariñas , and R. C. Rodríguez Martín‐Doimeadios , “Critical Evaluation of the Potential of ICP‐MS‐Based Systems in Toxicological Studies of Metallic Nanoparticles,” Analytical and Bioanalytical Chemistry 416, no. 11 (2024): 2657–2676. 38329514 10.1007/s00216-024-05181-4 PMC11009754

[88] F. Quattrini , G. Berrecoso , J. Crecente‐Campo , and M. J. Alonso , “Asymmetric Flow Field‐Flow Fractionation as a Multifunctional Technique for the Characterization of Polymeric Nanocarriers,” Drug Delivery and Translational Research 11 (2021): 373–395. 33521866 10.1007/s13346-021-00918-5 PMC7987708

[89] J. D. Robertson , L. Rizzello , M. Avila‐Olias , et al., “Purification of Nanoparticles by Size and Shape,” Scientific Reports 6, no. 1 (2016): 27494. 27271538 10.1038/srep27494 PMC4897710

[90] Z. Chen , D. Wang , S. Gu , N. Wu , K. Wang , and Y. Zhang , “Size Exclusion Chromatography and Asymmetrical Flow Field‐Flow Fractionation for Structural Characterization of Polysaccharides: A Comparative Review,” International Journal of Biological Macromolecules 277 (2024): 134236. 39079564 10.1016/j.ijbiomac.2024.134236

[91] A. Bosch , J. Bott , N. Warfving , and J. Nolde , “Investigation on the Skin Penetration of Synthetic Amorphous Silica (SAS) Used in Cosmetic Products,” Toxicology Letters 399 (2024): 80–104. 37541533 10.1016/j.toxlet.2023.07.016

[92] Y. Lai , L. Dong , X. Sheng , J. Chao , S. Yu , and J. Liu , “Monitoring the Cd^2+^ Release From Cd‐Containing Quantum Dots in Simulated Body Fluids by Size Exclusion Chromatography Coupled With ICP‐MS,” Analytical and Bioanalytical Chemistry 414, no. 18 (2022): 5529–5536. 35212781 10.1007/s00216-022-03976-x

[93] K. Ben‐Jeddou , M. Bakir , M. S. Jiménez , M. T. Gómez , I. Abad‐Álvaro , and F. Laborda , “Nanosilver‐Based Materials as Feed Additives: Evaluation of Their Transformations Along In Vitro Gastrointestinal Digestion in Pigs and Chickens by Using an ICP‐MS Based Analytical Platform,” Analytical and Bioanalytical Chemistry 416, no. 16 (2024): 3821–3833. 38777876 10.1007/s00216-024-05323-8 PMC11180633

[94] E. Ban , Y. S. Yoo , and E. J. Song , “Analysis and Applications of Nanoparticles in Capillary Electrophoresis,” Talanta 141 (2015): 15–20. 25966374 10.1016/j.talanta.2015.03.020

[95] U. Pyell , “Characterization of Nanoparticles by Capillary Electromigration Separation Techniques,” Electrophoresis 31, no. 5 (2010): 814–831. 20191544 10.1002/elps.200900555

[96] L. Trapiella‐Alfonso , G. Ramírez‐García , F. d'Orlyé , and A. Varenne , “Electromigration Separation Methodologies for the Characterization of Nanoparticles and the Evaluation of Their Behaviour in Biological Systems,” TrAC Trends in Analytical Chemistry 84 (2016): 121–130.

[97] A. R. Timerbaev , K. Pawlak , S. S. Aleksenko , L. S. Foteeva , M. Matczuk , and M. Jarosz , “Advances of CE‐ICP‐MS in Speciation Analysis Related to Metalloproteomics of Anticancer Drugs,” Talanta 102 (2012): 164–170. 23182589 10.1016/j.talanta.2012.07.031

[98] J. Sikorski , M. Drozd , and M. Matczuk , “Red Flags and Adversities on the Way to the Robust CE‐ICP‐MS/MS Quantitative Monitoring of Self‐Synthesized Magnetic Iron Oxide(II, III)‐Based Nanoparticle Interactions With Human Serum Proteins,” Molecules (Basel, Switzerland) 27, no. 23 (2022): 8442. 36500533 10.3390/molecules27238442 PMC9739417

[99] D. Baron , J. Rozsypal , A. Michel , et al., “Study of Interactions Between Carboxylated Core Shell Magnetic Nanoparticles and Polymyxin B by Capillary Electrophoresis With Inductively Coupled Plasma Mass Spectrometry,” Journal of Chromatography A 1609 (2020): 460433. 31427136 10.1016/j.chroma.2019.460433

[100] J. Kruszewska , J. Sikorski , J. Samsonowicz‐Górski , and M. Matczuk , “A CE‐ICP‐MS/MS Method for the Determination of Superparamagnetic Iron Oxide Nanoparticles Under Simulated Physiological Conditions,” Analytical and Bioanalytical Chemistry 412, no. 29 (2020): 8145–8153. 32968852 10.1007/s00216-020-02948-3 PMC7584539

[101] A. Wróblewska and M. Matczuk , “First Application of CE‐ICP‐MS for Monitoring the Formation of Cisplatin Targeting Delivery Systems With Gold Nanocarriers,” Electrophoresis 41, no. 5–6 (2020): 394–398. 31976562 10.1002/elps.201900438

[102] C. Adelantado , A. Ríos , and M. Zougagh , “A New Nanometrological Strategy for Titanium Dioxide Nanoparticles Screening and Confirmation in Personal Care Products by CE‐spICP‐MS,” Talanta 219 (2020): 121385. 32887088 10.1016/j.talanta.2020.121385

[103] C. M. Maguire , M. Rösslein , P. Wick , and A. Prina‐Mello , “Characterisation of Particles in Solution—A Perspective on Light Scattering and Comparative Technologies,” Science and Technology of Advanced Materials 19, no. 1 (2018): 732–745. 30369998 10.1080/14686996.2018.1517587 PMC6201793

[104] M. R. Moser and C. A. Baker , “Taylor Dispersion Analysis in Fused Silica Capillaries: A Tutorial Review,” Analytical Methods 13, no. 21 (2021): 2357–2373. 33999088 10.1039/d1ay00588j

[105] F. d'Orlyé , A. Varenne , and P. Gareil , “Determination of Nanoparticle Diffusion Coefficients by Taylor Dispersion Analysis Using a Capillary Electrophoresis Instrument,” Journal of Chromatography A 1204, no. 2 (2008): 226–232. 18718601 10.1016/j.chroma.2008.08.008

[106] A. Degasperi , L. Labied , C. Farre , et al., “Probing the Protein Corona of Gold/Silica Nanoparticles by Taylor Dispersion Analysis‐ICP‐MS,” Talanta 243 (2022): 123386. 35313133 10.1016/j.talanta.2022.123386

[107] I. Strzeminska , C. Factor , J. Jimenez‐Lamana , et al., “Comprehensive Speciation Analysis of Residual Gadolinium in Deep Cerebellar Nuclei in Rats Repeatedly Administered With Gadoterate Meglumine or Gadodiamide,” Investigative Radiology 57, no. 5 (2022): 283–292. 35066532 10.1097/RLI.0000000000000846 PMC9855751

